# Insights into the capability of the lignocellulolytic enzymes of *Penicillium* *parvum* 4-14 to saccharify corn bran after alkaline hydrogen peroxide pretreatment

**DOI:** 10.1186/s13068-023-02319-x

**Published:** 2023-05-11

**Authors:** Liangkun Long, Wei Wang, Zhen Liu, Yuanxin Lin, Jing Wang, Qunying Lin, Shaojun Ding

**Affiliations:** 1 Jiangsu Co-Innovation Center for Efficient Processing and Utilization of Forest Resources, College of Chemical Engineering, Nanjing Forestry University, Nanjing, 210037 China; 2 Jiangsu Provincial Key Lab for the Chemistry and Utilization of Agro-Forest Biomass, Nanjing Forestry University, Nanjing, 210037 China; 3 Nanjing Institute for the Comprehensive Utilization of Wild Plants, Nanjing, 211111 China

**Keywords:** Corn bran arabinoxylan, Carbohydrate-active enzymes, *Penicilliumparvum*, Genome, Secretome

## Abstract

**Background:**

Corn bran is a major agro-industrial byproduct from corn starch processing. It contains abundant arabinoxylan that can be converted into value-added chemicals via biotechnology. Corn bran arabinoxylan (CBAX) is one of the most recalcitrant xylans for enzymatic degradation due to its particular heterogeneous nature. The present study aimed to investigate the capability of the filamentous fungus *Penicillium* *parvum* 4-14 to enzymatically saccharify CBAX and reveal the fungal carbohydrate-active enzyme (CAZyme) repertoire by genome sequencing and secretome analysis.

**Results:**

CBAX1 and CBAX2 with different branching degrees, together with corn bran residue (CBR) were generated from corn bran after alkaline hydrogen peroxide (AHP) pretreatment and graded ethanol precipitation. The protein blends E_CBAX1, E_CBAX2, and E_CBR were produced by the fungus grown on CBAX1, CBAX2, or CBR, respectively. Under the optimal conditions, E_CBAX1 released more than 80% xylose and arabinose from CBAX1 and CBAX2. Almost complete saccharification of the arabinoxylans was achieved by combining E_CBAX1 and a commercial enzyme cocktail Cellic®CTec3. Approximately 89% glucose, 64% xylose, and 64% arabinose were liberated from CBR by E_CBR. The combination of E_CBR with Cellic®CTec3 enhanced the saccharification of CBR, with conversion ratios of 97% for glucose, 81% for xylose, and 76% for arabinose. A total of 376 CAZymes including plentiful lignocellulolytic enzymes were predicted in *P.* *parvum* based on the fungal genomic sequence (25.8 Mb). Proteomic analysis indicated that the expression of CAZymes in *P.* *parvum* varied between CBAX1 and CBR, and the fungus produced complete cellulases, numerous hemicellulases, as well as high levels of glycosidases under the culture conditions.

**Conclusions:**

This investigation disclosed the CAZyme repertoire of *P.* *parvum* at the genomic and proteomic levels, and elaborated on the promising potential of fungal lignocellulolytic enzymes upon saccharification of corn bran biomass after AHP pretreatment.

**Supplementary Information:**

The online version contains supplementary material available at 10.1186/s13068-023-02319-x.

## Background

Corn (*Zea* *mays* L.) is one of the four crops with the highest production and the largest cultivated area worldwide [1], and the global production of the cereal crop has reached 1.2 billion metric tons per year (https://www.statista.com). Corn bran is the outermost layer of the corn kernel and a plentiful byproduct of the corn flour industry, accounting for 6–9% of the corn grain weight [2]. After removing starch, corn bran consists mainly of hemicellulose (arabinoxylan, up to 47%), followed by cellulose (20–25%) and lignin (about 10%) [2, 3]. Owing to its high arabinoxylan content, corn bran is an attractive raw material for the production of pentose (xylose and arabinose), which can be converted into high-value chemicals or biofuels (e.g., xylitol, arabitol, lactic acid, ethanol, and lipid) through microbial fermentation in the bio-refining industry [4]. Enzymatic conversion of (hemi-)cellulose into fermentable sugars is a promising approach with multiple advantages, including mild reaction conditions, high specificity, environmental friendliness, and avoidance of the formation of inhibitory compounds or toxic byproducts, which impede the downstream fermentation process [5, 6]. The development of enzymatic technologies for saccharifying various plant xylans has received specific attention for the complete utilisation of lignocellulosic biomasses [7–9]. Notably, the structure of xylan changes among different plants, even in different parts of the same plant [10, 11]. Corn bran arabinoxylan (CBAX) is one of the most complex and recalcitrant plant xylans, and enzymatic degradation of the substrate is still a challenge [10, 12].

CBAX consists of a backbone of β-1,4-d-xylopyranosyl (Xyl*p*) units decorated with diverse side chains involving α-l-arabinofuranosyl (Ara*f*) residues at *O*-3 and/or *O*-2 positions, 4-*O*-methyl-α-d-glucuronic acid (m-GlcA) at *O*-2 position, and phenolic or acetyl groups on Ara*f* or Xyl*p* residues (Additional file 1: Fig. S1) [13]. The ratio of arabinose to xylose (*A/X*) in CBAX ranges from 0.46 to 0.74, depending on the material source (genotype) and extraction method [14, 15]. The high A/X ratio leads to a barrier to the degradation of CBAX by pure endo-xylanases belonging to glycoside hydrolase (GH) families 10 or 11, which require two (for GH10) or three (for GH11) consecutive unsubstituted Xyl*p* residues to attack the main chain [10]. Notably, some special oligosaccharide side chains typically containing the α-l-galactopyranosyl-(1,2)-β-d-Xyl*p*-(1,2)-5-*O*-*trans*-feruloyl-l-Ara*f* (FAXG) structure (Additional file 1: Fig. S1) have been identified in CBAX [16–18]. These large side chains in arabinoxylan elevate the resistance to degrading enzymes [19].

Complete saccharification of heteroxylans requires the synergistic action of multiple enzymes, which are divided into main-chain degrading enzymes and side-chain degrading (debranching) enzymes [20]. The main-chain enzymes include endo-xylanases (GH10, 11, and 30) and β-d-xylosidases (GH3 and 43) [20]. The debranching enzymes are mainly α-l-arabinofuranosidase (GH43, 51, 54, and 62), α-glucuronidase (GH67 and 115), and acetylxylan esterase (CE1 and 16) [10]. Other enzymes such as α-galactosidase (GH27, 36, and 95) acting on galactose residues and α-xylosidase (GH31) may be required to improve the degradation of plant xylans with complex structures (e.g., CBAX) [10, 19]. In addition, some lytic polysaccharide monooxygenases (LPMOs) in the auxiliary activity (AA) families 9 and 14 displayed positive roles in the biodegradation of xylans in previous studies [20–22]. Considering the limited saccharification of CBAX by several enzyme mixtures and/or commercial enzyme cocktails [12, 23], seeking new enzyme cocktails is one of the possible approaches to realise the highly efficient conversion of complex arabinoxylans into fermentable sugars.

From an industrial point of view, filamentous fungi are the most popular enzymatic arsenals for the deconstruction of plant biomass because of their strong ability to produce extracellular enzymes [20]. Commercial (hemi-)cellulases are typically produced by *Trichoderma* and *Aspergillus* species. For example, *Trichoderma* *reesei* (synonym: *Hypocrea* *jecorina*) has been developed as the most famous producer of cellulases [24], and *Aspergillus* *niger* is used industrially to produce hemicellulases [25]. Recent studies have shown that the two closely related genera *Penicillium* and *Talaromyces* are promising sources of hemicellulases [26]. Particularly, the carbohydrate-active enzymes (CAZymes) from *Penicillium* species exhibited different characteristics from those of *Trichoderma* and *Aspergillus* [27, 28].

The soil fungus *Penicillium* *parvum* (synonym: *Eupenicillium* *parvum*) 4-14 is a valuable decomposer of corn bran [29], and some lignocellulolytic enzymes of the fungus have been functionally characterised in our previous studies [30–33]. The current study aimed to exhaustively evaluate the capacity of the fungal lignocellulolytic enzymes to saccharify the soluble and insoluble fractions of corn bran after alkaline hydrogen peroxide (AHP) pretreatment. The enzyme production, activity composition, and optimal catalysis conditions on soluble hemicelluloses and solid fraction of AHP-pretreated corn bran were reported in this study. The properties of the fungal CAZymes were elucidated using genomic sequencing in combination with secretome analysis.

## Results

### Chemical composition of corn bran before and after AHP pretreatment

As shown in Table 1, the destarched corn bran consisted of 25.8% glucose, 27.1% xylose, 16.1% arabinose, 2.4% galactose, and 10.9% lignin. After AHP pretreatment and graded ethanol precipitation, the yields of CBAX1, CBAX2 and corn bran residue (CBR) were 24.7%, 19.2% and 24.5%, respectively. CBAX1 and CBAX2 were mainly composed of xylose (41.0% and 43.5%) and arabinose (22.5% and 30.3%), respectively, followed by glucose (11.7% and 3.4%), and galactose (4.6% and 3.4%). CBR contained 57.0% glucose, 18.5% xylose, 9.1% arabinose, 0.8% galactose, and 1.2% lignin. The degree of Ara*f* substitution of CBAX2 was higher than that of CBAX1 and CBR, which was confirmed by the *A*/*X* ratios in Table 1.

**Table 1:** The contents (%, w/w) of carbohydrates and lignin in corn bran before and after AHP pretreatment

Substrate	Glucose	Xylose	Arabinose	Galactose	Lignin	*A*/*X*	Yield (%)
Raw material	25.8 ± 0.7	27.1 ± 1.4	16.1 ± 0.5	2.4 ± 0.2	10.9 ± 0.6	0.59	100
CBAX1	11.7 ± 0.8	41.0 ± 2.2	22.5 ± 1.2	4.6 ± 0.1	–	0.55	24.7
CBAX2	3.4 ± 0.3	43.5 ± 4.1	30.3 ± 0.8	3.4 ± 0.2	–	0.70	19.2
CBR	57.0 ± 0.2	18.5 ± 0.3	9.1 ± 0.1	0.8 ± 0.1	1.2 ± 0.1	0.49	24.5

Corn bran arabinoxylan (CBAX) 1 and 2 were extracted from destarched corn bran by AHP pretreatment and graded ethanol precipitation, as described in the “Methods” section. CBR, corn bran residue; A/X, ratio of arabinose to xylose; -, no analysis. The yield (%) indicates the weight ratio of the substrate to the raw material. The data represent the mean of three independent measurements

### Extracellular proteins production by *P. parvum* on different carbon sources

*P.* *parvum* 4-14 was grown normally in modified Mandels’ medium, with CBAX1, CBAX2, or CBR as the sole carbon source. After 8 days of fungal growth, the protein concentrations in the supernatants of CBAX1, CBAX2, and CBR media were 0.57 mg/mL, 0.61 mg/mL and 1.12 mg/mL, respectively (Table 2). The amount of fungal extracellular proteins induced by CBR was nearly twofold that induced by CBAX1 or CBAX2, suggesting that more enzymes were required for the fungus to utilise the insoluble substrate. After salting-out and dialysis treatment, the concentrations of these crude proteins increased by 5.0- to 6.3-fold (Table 2). These protein (enzyme) blends produced by the fungus in CBAX1, CBAX2, and CBR media were marked as E_CBAX1, E_CBAX2, and E_CBR, respectively.

**Table 2:** Extracellular protein production by *P.* *parvum* 4-14 in liquid fermentation with different carbon sources

Carbon source	Protein concentration (mg/mL)		DCW (mg/mL)	Protein yield (mg/g DCW)
	Original	After concentration		
CBAX1	0.57 ± 0.10	3.58 ± 0.30	4.81 ± 0.42	117.8 ± 14.7
CBAX2	0.61 ± 0.04	3.07 ± 0.45	5.01 ± 0.37	122.5 ± 12.7
CBR	1.12 ± 0.19	5.79 ± 0.61	-	-

Fungal fermentation was carried out in 50 mL of 2 × Mandels' medium with 2% carbon source, and at 28 °C and 200 rpm for 8 days. For each group, 45 mL of fermentation supernatant was concentrated to 4 mL protein solution by salting-out and dialysis treatments. CBAX1 or 2, corn bran arabinoxylan 1 or 2; CBR, corn bran residue; DCW, dry cell weight; -, no analysis. Protein yields were calculated using the values of protein weight in the fermentation supernatant

**Fig. 1:**
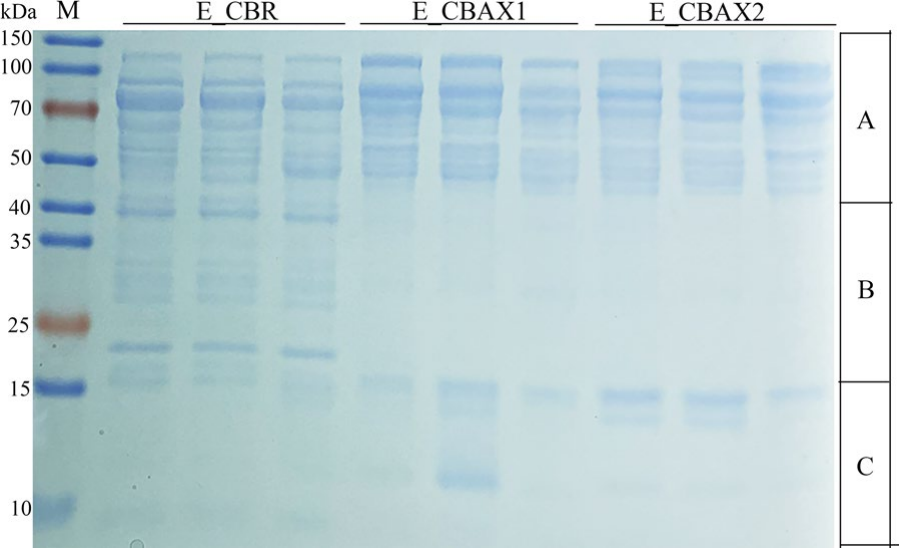
SDS-PAGE analysis of the secreted proteins by *P.* *parvum* 4-14 using different carbon sources. M, protein marker; E_CBR, extracellular proteins (enzymes) produced by the fungus using corn bran residue as carbon source; E_CBAX1 (or 2), extracellular proteins (enzymes) produced by the fungus using corn bran arabinoxylan 1 (or 2) as carbon source. About 32 µg of protein sample was loaded in each lane for electrophoresis on a 12% SDS-PAGE gel

Sodium dodecyl sulphate-polyacrylamide gel electrophoresis (SDS-PAGE) analysis showed that E_CBAX1 and E_CBAX2 had similar band compositions, which were distinctly different from that of E_CBR (Fig. 1). For E_CBAX1 and E_CBAX2, the protein bands intensities on the gel were highest in region A, followed by region C, and the lowest were in region B. The protein bands of E_CBR appeared intensely in regions A and B, and a few of them existed in region C. The band composition of E_CBAX1 or E_CBAX2 exhibited the most obvious difference from that of the E_CBR group in region B of the SDS-PAGE (Fig. 1).

### Enzymatic activities of the protein blends

Cellulase or hemicellulase activities against seven different substrates were detected in the protein blends: E_CBAX1, E_CBAX2, and E_CBR (Table 3). E_CBAX1 and E_CBAX2 possessed similar specific activities toward these substrates and showed higher hemicellulase activities and lower cellulase activities than the same amount of E_CBR. The specific activities of β-xylosidase, α-l-arabinofuranosidase, and α-galactosidase in E_CBAX1 and E_CBAX2 were approximately twice as high as those in E_CBR. Meanwhile, the CMCase activity and cellulase activity on phosphoric acid swollen cellulose (PASC) in E_CBR were 4.9- or 4.6-fold and 3.4- or 4.6-fold of those in E_CBAX1 or E_CBAX2, respectively (Table 3). The specific activity of xylanase in E_CBR showed a small (11–18%) decrement, compared with E_CBAX1 and E_CBAX2. The protein concentration of E_CBR was twofold that of E_CBAX1 (or E_CBAX2) (Table 2), indicating that the former contained more xylanase than the latter.

**Table 3:** Enzymatic activities of crude proteins produced by *P.* *parvum* 4-14 using different carbon sources

Enzyme activity (U/mg protein)	E_CBAX1	E_CBAX2	E_CBR
Xylanase	48.03 ± 1.28	52.39 ± 2.09	42.90 ± 1.24
β-Xylosidase	15.03 ± 0.03	14.67 ± 1.30	7.72 ± 0.08
α-Galactosidase	22.60 ± 0.11	21.11 ± 0.77	12.58 ± 1.39
α-Xylosidase	0.21 ± 0.00	0.30 ± 0.04	0.08 ± 0.00
α-l-Arabinofuranosidase	9.63 ± 0.44	9.88 ± 0.60	5.11 ± 0.17
CMCase	2.92 ± 0.04	3.06 ± 0.17	14.17 ± 0.14
Cellulase^a^	0.12 ± 0.00	0.09 ± 0.00	0.41 ± 0.00

E_CBAX1, E_CBAX2, and E_CBR, the enzyme blends produced by the fungus using CBAX1, CBAX2, or CBR as the sole carbon source, respectively. All enzyme blends were subjected to salting-out and dialysis treatments before use. The enzymatic reactions were carried out in 0.1 M sodium acetate buffer (pH 5.0) at 45 °C for 10 min, and the activities were determined as described in the “Methods” section.

^a^Enzyme activity was measured using PASC as a substrate. The data represent the averages of three independent assays

### Saccharification of extractable corn bran arabinoxylans

#### Selection of a protein blend for saccharification experiment

Saccharification of CBAX1 and CBAX2 was performed using E_CBAX1, E_CBAX2, or E_CBR at 0.625%, 1.25%, or 2.5% dosage, respectively. Under the same conditions, the amount of reducing sugars released from CBAX1 or CBAX2 by E_CBAX1 was higher than that by E_CBAX2 or E_CBR, especially at a low enzyme dosage (Additional file 2: Fig. S2a, b). Therefore, E_CBAX1 was chosen to continue the following experiments.

#### Optimal conditions for enzymatic hydrolysis

**Fig. 2:**
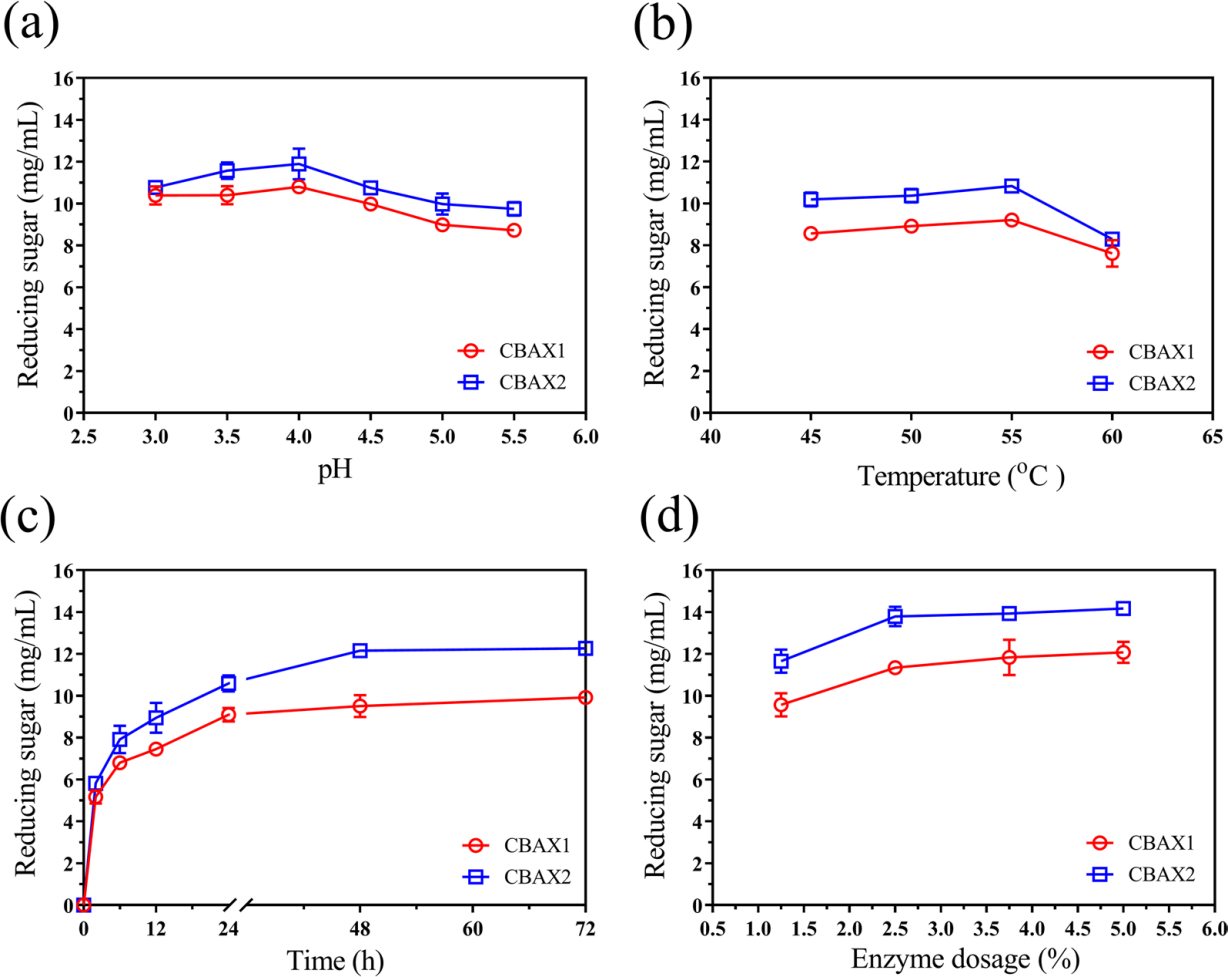
Effects of pH (**a**), temperature (**b**), reaction time (**c**) or enzyme dosage (**d**) on the saccharification of CBAX1 or CBAX2 by enzyme blend E_CBAX1. Except as indicated, the hydrolysis reaction was carried out in 200 μL sodium acetate buffer (0.1 M, pH 5.0) containing 4 mg CBAX1 (or CBAX2) and 50 μg (1.25%, g protein/g substrate) crude enzymes at 50 °C for 48 h. E_CBAX1, the enzyme blend produced by *P.* *parvum* 4-14 using CBAX1 as carbon source. Error bars represent standard deviations from three repeated measurements

Hydrolysis of CBAX1 and CBAX2 by E_CBAX1 was carried out under different conditions: pH, temperature, reaction time, enzyme dosage, and with or without additional metal ions (1 mM). The enzyme blend released the maximum level (10.8 mg/mL or 11.9 mg/mL) of reducing sugars from CBAX1 or CBAX2 at pH 4.0. Changing the pH value to 3.5, 3.0, or 4.5 resulted in a slight reduction (less than 10%) of the reducing sugars released by enzyme hydrolysis. At a higher pH value of 5.0 or 5.5, the amount of reducing sugar released by E_CBAX1 was reduced by 16–19% (Fig. 2a). The optimal temperature for enzymatic hydrolysis of the two substrates was 55 °C. A higher temperature (60 °C) led to a 17% or 23% decrement in the released reducing sugars from CBAX1 or CBAX2, respectively (Fig. 2b). The amounts of sugars liberated from the substrates by E_CBAX1 did not change after supplementation with Mg^2+^, Ca^2+^, Co^2+^, Zn^2+^ or EDTA, and had a slight decrement in the presence of Mn^2+^ (Additional file 2: Fig. S3).

The time course of enzymatic hydrolysis showed that reducing sugars were rapidly released from the substrates in the initial 24 h. The release gradually slowed from 24 to 48 h, and reached the highest level of reducing sugars when incubated for 72 h (Fig. 2c). Under the hydrolysis conditions, 1.25% protein dosage released 9.6 mg/mL or 11.6 mg/mL of reducing sugars from CBAX1 or CBAX2, respectively. Doubling the enzyme dosage increased the liberation of reducing sugars to 11.3 mg/mL from CBAX1 or 13.8 mg/mL from CBAX2. A slow increase in the released reducing sugars was observed when the protein usage was elevated to 3.75% and 5.0%. The maximum liberation of reducing sugars from CBAX1 and CBAX2 were 12.1 mg/mL and 14.2 mg/mL, respectively (Fig. 2d).

#### Analysis of monosaccharide liberation during the enzymatic hydrolysis

**Fig. 3:**
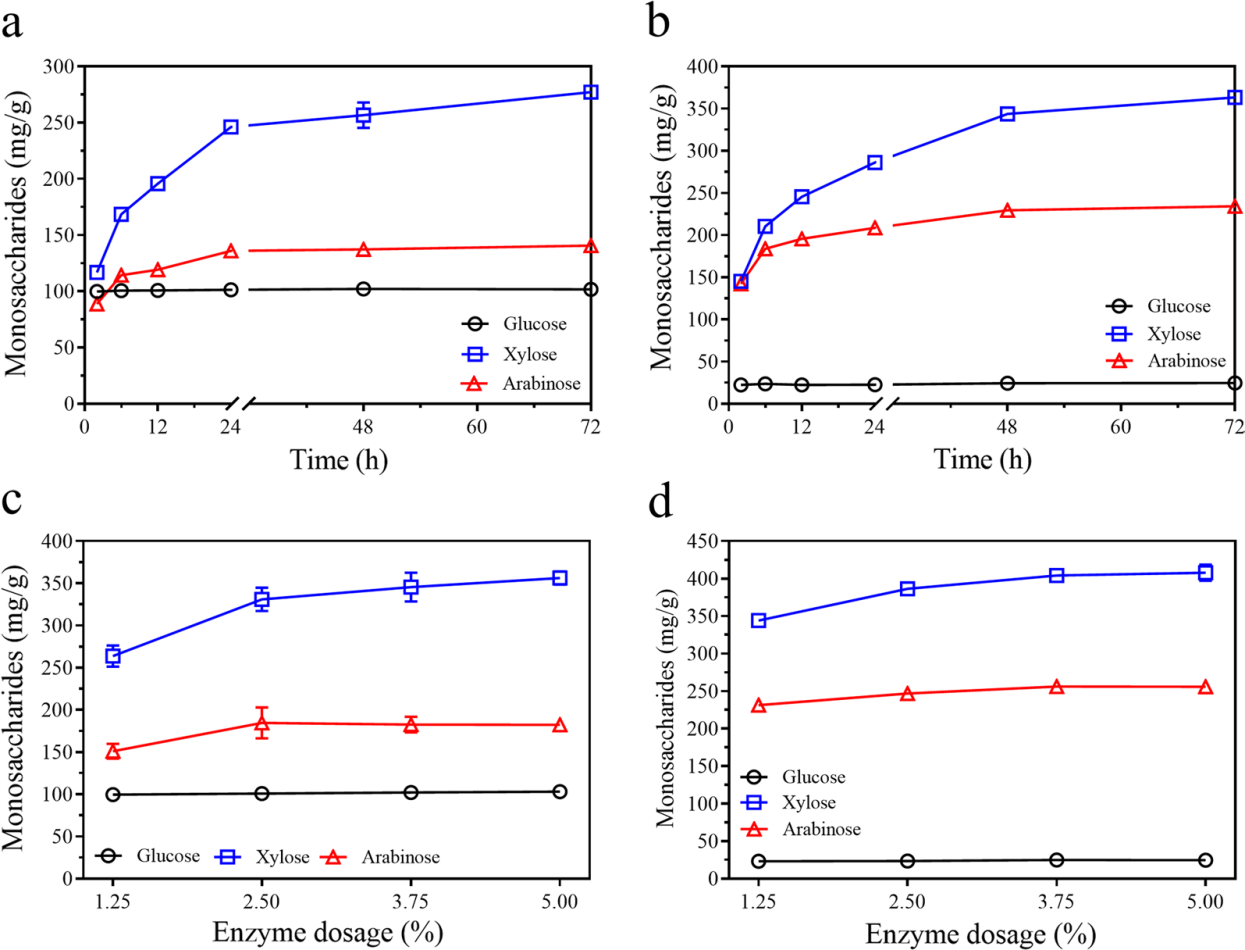
Analysis of monosaccharide released from CBAX by enzyme blend E_CBAX1. **a**, **b** Time-course of liberation of monosaccharide from CBAX1 (**a**) or CBAX2 (**b**) during the enzymatic hydrolysis. **c**, **d** Monosaccharide liberation from CBAX1 (**c**) or CBAX2 (**d**) by the enzyme blend at different dosages (0.12–5.0%, g protein/g substrate). Except as indicated, the hydrolysis reaction was carried out in 200 μL sodium acetate buffer (0.1 M, pH 4.0) containing 4 mg CBAX1 (or CBAX2) and 50 μg (w/w, 1.25%) crude enzymes at 50 °C for 48 h. E_CBAX1, the enzyme blend produced by *P.* *parvum* 4-14 using CBAX1 as the sole carbon source. Error bars represent standard deviations from three repeated measurements

Quantitative analysis of monosaccharides indicated that 116.8 mg/g (conversion ratio 28.5%) or 145.4 mg/g (33.5%) xylose, 88.8 mg/g (39.4%) or 142.3 mg/g (46.9%) arabinose, and 99.9 mg/g (84.0%) or 22.4 mg/g (71.7%) glucose were released from CBAX1 or CBAX2 after 2 h of enzymatic hydrolysis, respectively (Fig. 3a, b, Additional file 3: Table S1). The amounts of xylose and arabinose, but not glucose, released from the substrates by E_CBAX1 increased quickly from 2 to 24 h for CBAX1, or 2 to 48 h for CBAX2, and then slowly. After 72 h of incubation, the liberations of xylose, arabinose, and glucose from CBAX1 or CBAX2 reached 277.1 mg/g (67.7%), 140.8 mg/g (62.5%) and 101.7 mg/g (85.5%), or 363.3 mg/g (83.6%), 234.2 mg/g (77.3%) and 24.7 mg/g (79.1%), respectively (Additional file 3: Table S1). For each substrate, the conversion ratio of xylose was lower than that of arabinose in the initial hydrolysis stage (2–12 h), and the former was close to (24 and 48 h) and higher (72 h) than the latter in a longer reaction time. Meanwhile, the A/X values of the hydrolysates gradually decreased from 0.76 to 0.51 for CBAX1, or from 0.98 to 0.64 for CBAX2, with the extension of reaction time (Additional file 3: Table S1). These data indicated that the release of arabinose from each substrate was faster than that of xylose during enzymatic hydrolysis, suggesting that removing the Ara*f* substitute from the Xyl*p* backbone was requisite for the hydrolysis of xylanases. The conversion ratio of pentose by the enzyme blend on CBAX1 (*A*/*X* 0.55) was lower than that on CBAX2 (*A*/*X* 0.7), particularly when a low enzyme dosage was used (Additional file 3: Table S2). Increasing the enzyme dosage improved the release of xylose and arabinose from substrates (Fig. 3c, d). The highest amounts of released xylose, arabinose, and glucose from CBAX1 or CBAX2 by E_CBAX1 (5.0% dosage) were 356.3 mg/g (87.0%), 182.2 mg/g (80.8%) and 103.2 mg/g (86.7%), or 407.7 mg/g (93.8%), 255.8 mg/g (84.4%) and 24.8 mg/g (79.4%), respectively (Additional file 3: Table S2).

#### Enhancement of CBAX saccharification by E_CBAX1 in combination with CTec3

**Fig. 4:**
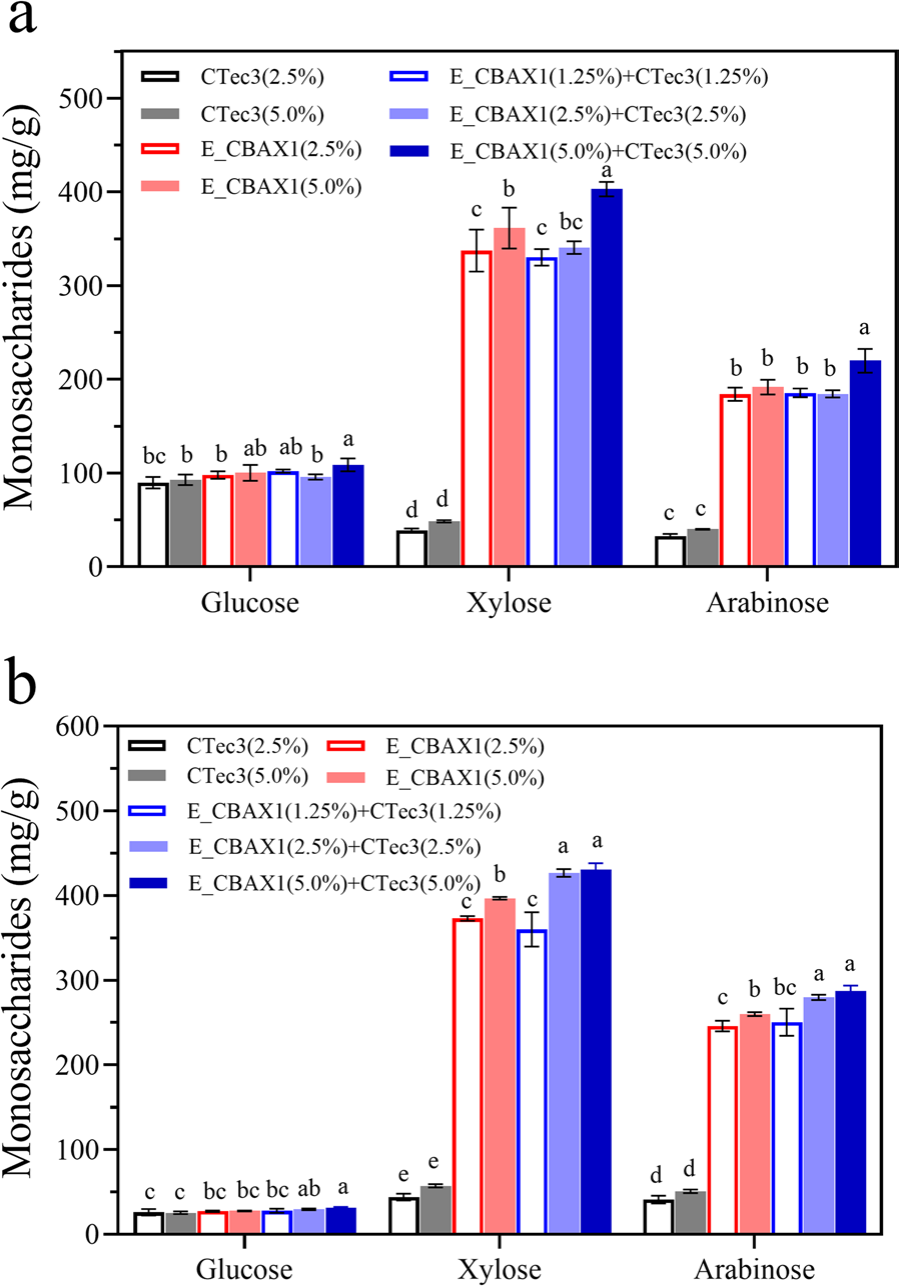
Saccharification of CBAX1 (**a**) or CBAX2 (**b**) by the enzyme blends E_CBAX1 and/or CTec3. The enzymatic hydrolyses were carried out in a 2-mL tube containing 0.2 mL sodium acetate buffer (0.1 M, pH 4.0), 4 mg substrate and the corresponding enzymes, at 50 °C and 800 rpm for 3 days. The released monosaccharides were quantified by HPLC analysis. CTec3, commercial *T.* *reesei* cellulase Cellic^®^ CTec3; E_CBAX1, the enzyme blend produced by *P.* *parvum* 4-14 using CBAX 1 as carbon source. The data are the averages from three repeated measurements. In the same group, different letters above the error bars indicate significant differences between treatments (LSD test, *P* < 0.05)

The commercial (hemi-)cellulase cocktail Cellic®CTec3 (CTec3) showed a low capability on saccharification of CBAX1 and CBAX2 (Fig. 4a, b). The conversion ratio of xylose or arabinose on the two substrates by CTec3 at a high (5.0%) dosage was less than 20%, which was much lower than that (over 85%) by E_CBAX1 with the same dosage (Additional file 3: Tables S3, S4). The majority of the glucose in each substrate was released by CTec3 under the experimental conditions. Of note, saccharification of the extracted arabinoxylans was enhanced by the co-action of E_CBAX1 and CTec3 (Fig. 4a, b). Almost all polysaccharides in the substrates were converted into monosaccharides by using high dosages (5.0%) of E_CBAX1 and CTec3. After the enzymatic hydrolysis, 402.9 mg/g (98.4%) xylose, 219.9 mg/g (97.5%) arabinose, and 108.8 mg/g (91.4%) glucose were liberated from CBAX1 (Additional file 3: Table S3). For CBAX2, the amounts of released three monosaccharides by the same enzyme combination reached 429.1 mg/g (98.6%), 287.3 mg/g (94.8%), and 30.9 mg/g (98.9%), respectively (Additional file 3: Table S4).

#### Saccharification of corn bran residue

**Fig. 5:**
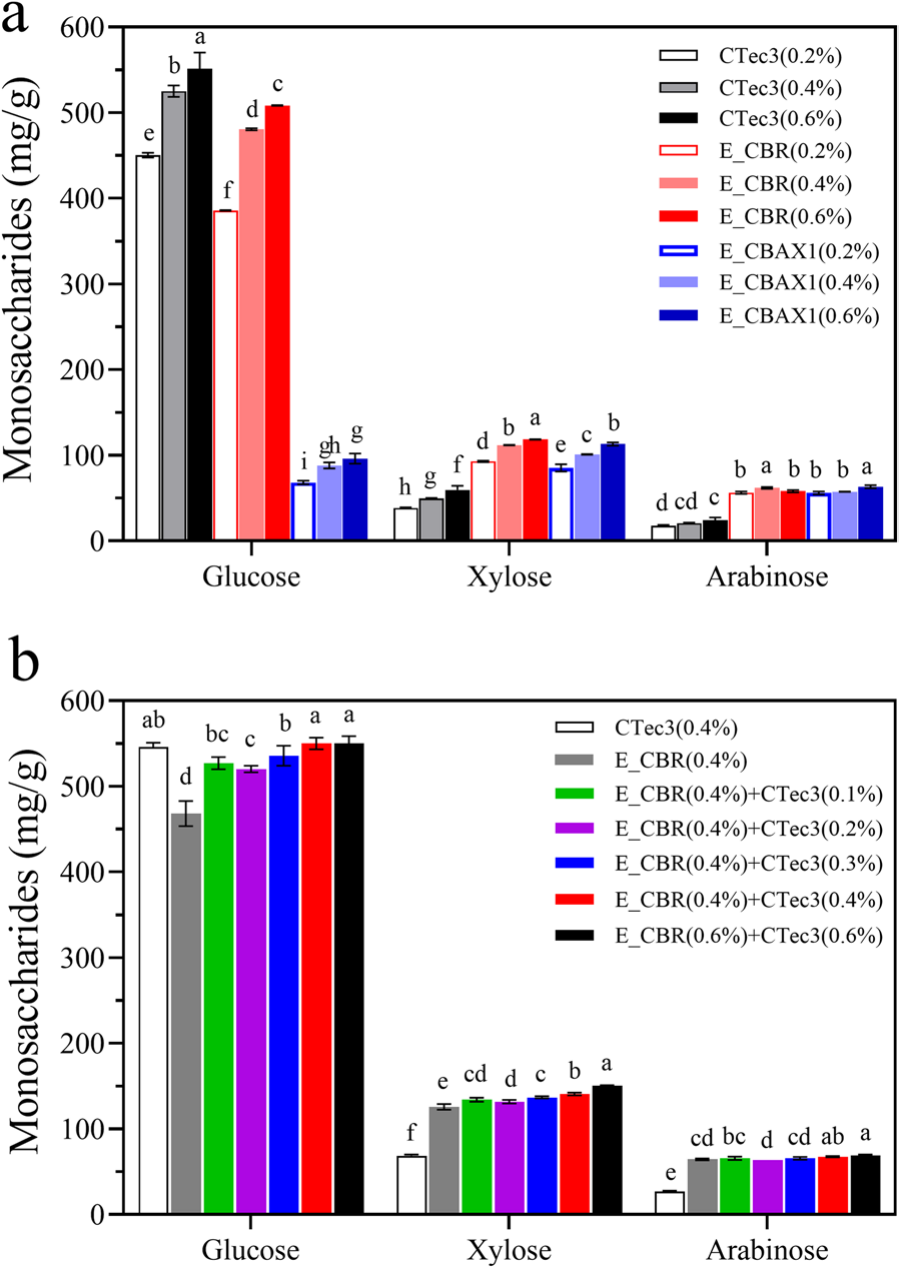
Saccharification of CBR by the enzyme blends E_CBR, E_CBAX1 and CTec3 separately (**a**), or the enzyme combinations of E_CBR and CTec3 (**b**). The enzymatic hydrolyses were carried out in a 2-mL tube containing 1 mL sodium acetate buffer (0.1 M, pH 4.5), 100 mg substrate and the corresponding enzymes, at 50 °C and 1200 rpm for 4 days. The released monosaccharides were quantified by HPLC analysis. CTec3, commercial cellulase Cellic^®^ CTec3; E_CBAX1 and E_CBR, the enzyme blends produced by *P.* *parvum* 4-14 using CBAX1 or CBR as carbon source, respectively. The data are the averages from three repeated measurements. In the same group, different letters above the error bars indicate significant differences between treatments (LSD test, *P* < 0.05)

Hydrolysis of CBR with 10% solid loading was performed using CTec3, E_CBAX1, or E_CBR at 0.2%, 0.4%, and 0.6% dosages, respectively. Among them, CTec3 exhibited the highest saccharification efficiency on the cellulose of CBR, and the lowest conversion ratio on the hemicellulose fraction of the substrate. The amounts of released glucose from the substrate by the low, medium, and high dosages of CTec3 were 451.7 mg/g (79.3%), 527.7 mg/g (92.7%), and 555.3 mg/g (97.5%), respectively (Fig. 5a; Additional file 3: Table S5). Meanwhile, the high dosage (0.6%) of CTec3 only released 59.5 mg/g (32.2%) xylose and 24.2 mg/g (26.7%) arabinose from CBR. Notably, the enzyme blend E_CBR displayed high efficiency upon the hydrolysis of cellulose as well as hemicellulose of CBR. The enzyme blend (0.6% dosage) released 508.4 mg/g (89.3%) glucose, 118.4 mg/g (64.2%) xylose, and 58.2 mg/g (64.1%) arabinose from CBR (Fig. 5a; Additional file 3: Table S5). The amounts of glucose, xylose, and arabinose released by E_CBR (0.6% dosage) were 0.92-fold, 1.98-fold, and 2.4-fold of those released by the same dosage of CTec3, respectively. Compared with E_CBR, E_CBAX1 showed a similar conversion ratio on the hemicellulose of CBR and a much lower saccharification efficiency on the cellulose of the substrate (Fig. 5a; Additional file 3: Table S5).

Saccharification of CBR was improved by E_CBR in combination with CTec3, which was reflected in the fact that the total amount of monosaccharides released by the combined enzymes was higher than that released by E_CBR or CTec3 alone (Fig. 5b; Additional file 3: Table S6). Compared with E_CBR (0.4% dosage), the amounts of released glucose, xylose, and arabinose from the substrate by E_CBR combined with CTec3 (0.4% dosage each) were increased by 18%, 12%, and 5%, respectively. Increasing the dosage of each enzyme blend to 0.6% resulted in the liberation of 554.1 mg/g (97.3%) glucose, 150.2 mg/g (81.4%) xylose, and 69.0 mg/g (76.1%) arabinose from the solid fraction of AHP-pretreated corn bran (Fig. 5b; Additional file 3: Table S6).

#### General properties and functional annotation of *P. parvum* genome

Based on the data from the PacBio Sequel platform as well as the Illumina NovaSeq PE150 platform (Additional file 4: Table S7), the 25.8 Mb genome (BioProject PRJNA901676, GenBank accession number JAPJNL000000000) of the *P.* *parvum* isolate 4-14b was assembled into 8 contigs with an N50 value of 3.92 Mb and an overall GC content of 52.1%. The size of the fungal genome is smaller than that of many lignocellulose-degrading fungi, including *P.* *decumbens* (30.2 M), *T.* *reesei* (33.9 M), *A.* *niger* (33.9 M), and *A.* *oryzae* (36.5 M) (Additional file 4: Table S8). Searching the genome sequence against 758 Benchmarking Universal Single-Copy Orthologues (BUSCOs) from the fungal_odb9 dataset revealed that 97.7% of the fungal BUSCOs were complete, 1.2% were fragmented, and 1.1% were missed, suggesting that the assembly was relatively contiguous. The general properties of the fungal genome are listed in Table 4.

**Table 4:** General properties of *P.* *parvum* 4-14b genome

Genomic feature	Value
Genome size (Mb)	25.8
GC content (%)	52.1
All protein-coding genes	8897
Secretory protein-coding genes	483
Coverage (fold)	97 ×
Percentage repeat rate	2.8
Gene average length (bp)	1461
Gene density (genes per Mb)	345.2
Average number of exons per gene	3.0
tRNA gene	147

In total, 8,897 protein-encoding genes were annotated in the fungal genome. Among them, 67.24% (5982), 90.15% (8021), 24.12% (2146), 93.19% (8291), 67.22% (5981), 37.72% (3356), 6.90% (614), and 4.23% (376) genes exhibited similarity (E-value less than 1e-5, coverage ≥ 40%, and identity ≥ 40%) to the annotated proteins in the Gene Ontology (GO), Kyoto Encyclopedia of Genes and Genomes (KEGG), Cluster of Orthologous Groups (COG), Non-Redundant (NR), Pfam, Swiss-Prot, Transporter Classification Database (TCDB), and CAZymes databases, respectively. Additionally, 146 cytochrome P450 proteins (genes), 484 secreted proteins (genes), and 35 secondary metabolism clusters (involving 475 genes) (Additional file 4: Table S9) were predicted to be present in the fungal genome. The deduced amino acid sequences of all the genes are listed in Additional file 5.

#### Abundant CAZyme-encoding genes in *P. parvum*

The fungal CAZyme repertoire was composed of 221 GHs (63 families), 14 carbohydrate esterases (CEs, 7 families), 3 polysaccharide lyases (PLs, 1 family), 44 auxiliary activities (AAs, 10 families), 94 glycosyltransferases (GTs, 30 families), and 28 carbohydrate-binding modules (CBMs, 11 families) (Table 5; Additional file 6: Table S10). The number of CAZymes (376) of *P.* *parvum* was close to that of *P.* *decumbens* (358) and *T.* *reesei* (387) and less than that of *A.* *niger* (475) or *A.* *oryzae* (518) (Additional file 7: Table S11). N-terminal signal peptides (SP) were predicted in 124 GHs (56.1%), 10 CEs (71.4%), 3 PLs (100%), and 19 AAs (43.2%) (Table 5; Additional file 6: Table S10), suggesting that they are extracellular proteins. In total, 82 proteins involved in the degradation of cellulose or hemicellulose were found in the predicted CAZyme repertoire of *P.* *parvum*. The number was close to that of *P.* *decumbens* (81) and higher than that of *T.* *reesei* (68) (Additional file 8: Table S12). Compared to *T.* *reesei,* *P.* *parvum* (also *P.* *decumbens*) possesses more hemicellulolytic enzymes. At least eight α-L-arabinofuranosidases were predicted in the genome of *P.* *parvum*, and only three were found in *T.* *reesei*. Three α-xylosidases (GH31) and two feruloyl esterases (CE1) were identified in *P.* *parvum*, and the two types of enzymes were deficient in *T.* *reesei.* Meanwhile, *T.* *reesei* had CE15 glucuronooyl esterase, GH74 xyloglucan-specific endo-β-d-1,4-glucanase, and GH115 α-glucuronidase/α-(4-*O*-methyl)-glucuronidase enzymes, which were not found in *P.* *parvum* (Additional file 8: Table S12).

**Table 5:** The number of CAZymes and non-CAZy proteins from *P.* *parvum* resulting from genome prediction and secretome analysis

Classification	Family	In genome		In secretome			
		All	With SP^a^	All	With SP	E_CBAX1	E_CBR
CAZymes	GH	221	124	108	92	100	107
	CE	14	10	6	5	5	6
	PL	3	3	2	2	1	2
	AA	44	19	3	3	2	3
	GT	94	0	0	0	0	0
	CBM^b^	28	21	17	14	16	16
	Sum	376	156	119	102	108	118
Non-CAZy proteins		8521	–	210	–	155	198

*GH* glycoside hydrolase, *CE* carbohydrate esterase, *PL* polysaccharide lyase, *AA* auxiliary activity, *GT* glycosyltransferase, *CBM* carbohydrate-binding module

^a^Prediction of signal peptide (SP) was performed using SignalP6.0 [71];

^b^CAZymes harbouring a CBM domain; –, no analysis

#### Comparison of *P. parvum* secretomes on different carbon sources

Using filter-aided sample preparation (FASP) and liquid chromatography–tandem mass spectrometry (LC–MS/MS) analysis, a total of 329 proteins were identified in the secretomes (E_CBAX1 and E_CBR) of *P.* *parvum*, accounting for 3.7% of the genome-predicted proteins (Additional file 9: Table S13). Among them, 119 (36.2%) proteins were identified as CAZymes, including 108 GHs, 6 CEs, 2 PLs, and 3 AAs (Table 5). These extracellular CAZymes have theoretical molecular weights (MWs) ranging from 22.1 kDa to 116.4 kDa, and the majority (85.7%) of them contain an N-terminal SP. The 210 (63.8%) non-CAZy proteins in the secretomes were composed of putative lipases, peptidases, oxidoreductases, permeases, and proteins with unknown functions. A total of 107 CAZy proteins (99 GHs, 5 CEs, 1 PLs, and 2 AAs) were commonly identified in E_CBAX1 and E_CBR. One putative α-1,3-glucanase belonging to GH71 family (PP00008) was only found in E_CBAX1. Eleven CAZymes were exclusively identified in E_CBR, including GH5 endo-β-1,4-mannanase (PP04925), GH18 chitinase (PP02488), GH31 α-glucosidase (PP07129), GH32 exo-inulinase (PP00628), GH55 exo-β-1,3-glucanase (01794), GH81 endo-β-1,3-glucanase (PP00639), GH132 β-glucosidase (SUN family) (PP04271), GH162 endo-β-1,2-glucanase (PP06039), CE1 feruloyl esterase (PP07504), PL1 pectate lyase (PP00847), and AA1 family laccase-like multicopper oxidase (PP01940) (Additional file 9: Table S13). In the fungal genome, 72 putative CAZy proteins (genes) distributed in 22 families were the main lignocellulolytic enzymes, of which 44 or 46 members appeared in E_CBAX1 or E_CBR, respectively. Among them, all members of GH6, 7, 10, 11, 27, 30, 67, or 131, and CE16 families were commonly produced by the fungus grown on CBAX1 or CBR (Additional file 10: Fig. S4). Detailed information on the proteins involved in cellulose or hemicellulose degradation is provided in Table 6.

**Table 6:** Summary of the identified (hemi-)cellulolytic enzymes in the secretomes of *P.* *parvum*

Protein ID	CAZy family	Description	Length (aa)	MW (kDa)	SP	Protein abundance (%)	
						E_CBAX1	E_CBR
Cellulolytic enzymes (17)							
PP01862	GH1	β-Glucosidase	562	63.1	Y	0.003	0.030
PP08205	GH1	β-Glucosidase	618	69.5	Y	0.014	0.017
PP00508	GH3	β-Glucosidase	862	93.5	Y	5.785	2.402
PP00690	GH3	β-Glucosidase	801	86.4	Y	0.025	0.012
PP07999	GH3	β-Glucosidase	732	77.7	Y	2.640	6.753
PP04992	GH3	β-Glucosidase	631	68.4	N	0.067	0.018
PP04712	GH3	β-Glucosidase	625	68.2	N	0.047	0.143
PP03978	GH5_5, CBM1	Endo-β-1,4-glucanase	436	47.1	Y	0.218	0.976
PP02987	GH5_5	Endo-β-1,4-glucanase	329	36.2	Y	0.034	1.376
PP07188	GH6	Cellobiohydrolase	387	41.0	Y	0.105	4.718
PP06287	GH7, CBM1	Cellobiohydrolase	527	55.4	Y	0.060	8.617
PP08806	GH7	Cellobiohydrolase	453	48.0	Y	0.078	0.424
PP07177	GH7	Endo-β-1,4-glucanase	421	44.9	Y	1.617	1.803
PP07505	GH12	Endo-β-1,4-glucanase	236	25.6	Y	0.146	3.762
PP03576	GH131, CBM1	Endo-β-1,4-glucanase	261	29.1	Y	0.017	0.322
PP08607	AA9	LPMO activity	249	26.3	Y	0.087	5.657
PP01505	AA9	LPMO activity	378	38.4	Y	0.007	0.032
Hemicellulolytic enzymes (33)							
PP02076	GH3	β-1,4-Xylosidase	793	86.4	Y	6.635	4.885
PP05440	GH3	β-1,4-Xylosidase	769	82.4	Y	13.712	4.782
PP08285	GH10	Endo-β-1,4-xylanase	326	35.1	Y	0.024	0.388
PP06205	GH10; CBM1	Endo-β-1,4-xylanase	404	43.1	Y	0.043	1.420
PP03584	GH11	Endo-β-1,4-xylanase	221	23.6	Y	0.513	1.452
PP05628	GH11, CBM1	Endo-β-1,4-xylanase	214	22.9	Y	1.045	0.490
PP00661	GH11	Endo-β-1,4-xylanase	210	22.1	Y	0.672	0.224
PP08282	GH30_7	Xylanase (4-Me-GlcA or GlcA-substituted xylan)	522	54.7	Y	0.172	0.225
PP01328	GH30_7, CBM1	Endo-β-1,4-xylanase	465	51.3	Y	2.583	2.990
PP08283	GH43_36, CBM1	α-l-Arabinofuranosidase	565	60.4	Y	0.079	0.469
PP03555	GH51	α-l-Arabinofuranosidase	636	69.7	Y	1.701	0.584
PP07776	GH54, CBM42	α-l-Arabinofuranosidase	503	52.0	Y	2.171	1.708
PP08284	GH62, CBM1	α-l-Arabinofuranosidase	325	35.7	Y	0.110	0.473
PP07503	GH62	α-l-Arabinofuranosidase	328	35.4	Y	0.035	0.865
PP04688	GH67	α-1,2-Glucuronidase	834	91.9	Y	0.285	0.473
PP00666	GH12	Xyloglucan-specific Endo-β-d-1,4-glucanase	249	26.4	Y	0.017	0.046
PP02109	GH31	α-Xylosidase	742	83.5	N	0.034	0.014
PP05558	GH2	β-Mannosidase	933	104.8	Y	0.296	0.119
PP04925	GH5_4	Endo-β-1,4-mannanase	592	63.7	Y	0.000	0.150
PP02075	GH29	α-l-Fucosidase	539	60.5	N	0.836	0.334
PP07138	GH95	α-l-Fucosidase	1017	115.1	N	0.018	0.020
PP04962	GH95	α-l-Fucosidase	404	44.1	Y	0.600	0.283
PP04963	GH95	α-l-Fucosidase	368	39.6	N	0.231	0.109
PP07518	GH27, CBM35	α-Galactosidase	560	60.5	N	0.413	0.126
PP06635	GH27, CBM13	α-Galactosidase	543	59.2	Y	0.144	0.081
PP00933	GH27	α-Galactosidase	470	51.6	Y	0.250	0.218
PP04893	GH27	α-Galactosidase	435	47.7	Y	3.362	0.838
PP00568	GH2	β-Galactosidase	644	71.5	Y	0.015	0.008
PP06827	GH2	Unknown	720	80.5	Y	0.047	0.074
PP05586	CE1	Feruloyl esterase	526	57.7	Y	0.015	0.014
PP07504	CE1	Feruloyl esterase	280	30.2	Y	0.000	0.019
PP03694	CE5	Acetyl xylan esterase	234	23.6	Y	0.702	0.817
PP02986	CE16	Acetyl xylan esterase	257	28.4	N	0.020	0.019

*LPMO* copper-dependent lytic polysaccharide monooxygenase, *MW* molecular weight, *SP* signal peptide, *Y* yes, *N* no

Protein abundance: the percentage of the target protein intensity to the total protein intensity in the sample

**Fig. 6:**
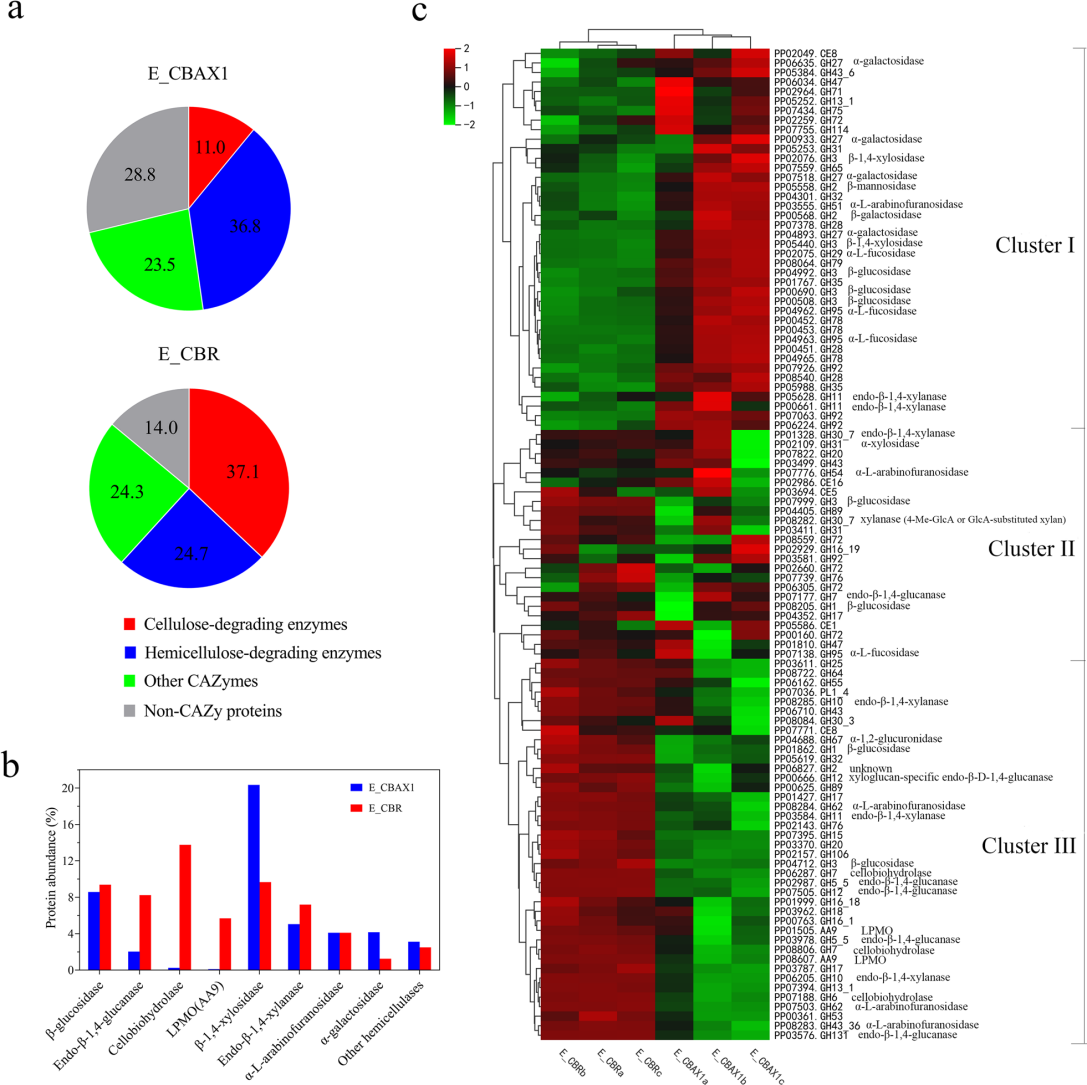
Comparison of CAZymes expression in the secretomes of *P.* *parvum* grown on different carbon sources. **a** Distribution of protein abundance (%) of the fungal secretomes. **b** Protein abundances of cellulose- and hemicellulose-degrading enzymes from the fungal secretomes. **c** Expression of CAZymes in two secretomes of *P.* *parvum.* E_CBAX1 or E_CBR, the secreted proteins by *P.* *parvum* 4-14 using CBAX1 or CBR as the sole carbon source, respectively. Protein abundance (%) represents the percentage of the LFQ intensity of target protein to the total LFQ intensity of proteins in the sample. The cluster heatmap of 104 CAZymes expressed in all samples was drawn by an online software (https://cloud.metware.cn), with Z-score normalised LFQ intensities (log2-transformed). The values shown are the averages of three biological replicates

Based on protein quantitative analysis, CAZymes were the major component of E_CBAX1 or E_CBR, accounting for 71.2% or 86.0% of the total protein abundance, respectively. For E_CBAX1, the protein abundance distribution was 36.8% for hemicellulose-degrading enzymes, 11.0% for cellulose-degrading enzymes, 23.5% for other CAZymes, and 28.8% for non-CAZy proteins. In contrast, E_CBR mainly contained cellulose-degrading enzymes (37.1%), followed by hemicellulose-degrading enzymes (24.7%) and other CAZymes (24.3%), and the least were non-CAZy proteins (14.0%) (Fig. 6a). Notably, abundant glycosidases, including β-xylosidase, β-glucosidase, α-galactosidase, and α-l-arabinofuranosidase, were produced by the fungus under the experimental conditions. Two GH3 β-1,4-xylosidases (PP02076 and PP05440) accounted for 20.3% and 9.7% of the total protein abundance in E_CBAX1 and E_CBR, respectively. The total abundances of the two GH3 β-glucosidases (PP00508 and PP07999) were 8.4% for E_CBAX1 and 9.2% for E_CBR. In addition, α-galactosidase (mainly protein PP04893) and α-l-arabinofuranosidase accounted for 4.2% and 4.1% of the total protein abundance of E_CBAX1, respectively (Table 6; Fig. 6b).

Hierarchical cluster analysis based on the relative abundance of 104 CAZymes showed that the secretomes of *P.* *parvum* were divided into two groups, which corresponded to the type of carbon source (Fig. 6c). According to their expression patterns, these CAZymes belong to three main clusters. Cluster I was composed of 40 proteins that showed higher relative abundance under the carbon source CBAX1 than under CBR. Four GH27 α-galactosidases, two GH3 β-1,4-xylosidase, and two GH11 endo-β-1,4-xylanase belong to this cluster. Cluster II contained 24 CAZy proteins, which had similar relative abundances between the two carbon sources. Cluster III comprised 40 CAZymes that had a higher relative abundance under CBR than under CBAX1. The majority of cellulolytic enzymes (e.g., three cellobiohydrolases belonging to GH7 or GH6 families, and two AA9 LPMOs), as well as several hemicellulolytic enzymes (e.g., two GH10 endo-β-1,4-xylanases and two GH62 α-l-arabinofuranosidases), existed in the group (Fig. 6c).

## Discussion

This study aimed to evaluate the saccharification capability of *P.* *parvum* lignocellulolytic enzymes on corn bran, particularly its hemicellulose fraction. Various pretreatment processes involving acid [34], alkali [35], autohydrolysis [36], and AHP [37] have been established to enhance the enzymatic hydrolysis of recalcitrant biomass. Of note, AHP treatment is a highly efficient approach for removing lignin in plant biomass through the strong oxidising power of H_2_O_2_ under alkaline condition [38, 39], and isolating hemicellulose with relatively complete structure from substrate by a dissolution mechanism [39]. Compared with a standard alkaline pretreatment, AHP process offers the advantages of mild reaction temperature (below 50 °C), environmental friendliness (low alkaline usage), and less formation of toxic byproducts [38, 39]. In the current study, AHP pretreatment dissolved the majority of the destarched corn bran and resulted in a CBR yield of 24.5%. A total of alkali-extractable arabinoxylans with 43.9% yield were recovered from the liquid fraction by graded ethanol precipitation. The contents of carbohydrates and lignin in the raw corn bran are consistent with the data in previous research [29]. A large amount of xylose and arabinose, as well as a small amount of galactose, were detected in CBAX1 and CBAX2 (Table 1). These substrates contained a minor amount of glucose, which could be explained as that the residual starch and/or xyloglucan in the raw materials were solubilised by the extraction solvents [15, 40]. CBR was mainly composed of cellulose and a certain amount of hemicellulose. CBAX2 had the highest *A*/*X* value (0.70), followed by CBAX1 (0.55), and insoluble arabinoxylan had the lowest *A*/*X* value in CBR (0.49). This is in agreement with the viewpoint that the distribution of Ara*f* substitutions on xylan backbone is not random, and xylan subfractions with varied branching degrees form in plant cell walls [41, 42]. The low branching degree of xylan in CBR facilitates tight binding with cellulose and avoids its removal by chemical treatment [41]. The branching degree affects the physical properties of xylan, including solubility, rigidity, and self-aggregation [41, 43], which may further influence the enzymatic digestibility of the substrate.

The synthesis of lignocellulosic enzymes by filamentous fungi is largely affected by carbon sources [44]. In order to stimulate enzyme production, *P.* *parvum* was grown in a chemically defined medium using CBAX1, CBAX2, or CBR as the sole carbon source. Under the induction of CBAX1 or CBAX2, the fungus produced protein blends (E_CBAX1 and E_CBAX2) with similar yields, enzymatic activities, and protein band compositions (Tables 2 and 3; Fig. 1). Compared with E_CBAX1 and E_CBAX2, the total amount of proteins (E_CBR) secreted by *P.* *parvum* doubled in the presence of CBR, suggesting that more enzymes were required for the fungus to destroy the solid substrate than soluble xylans. By comparing the protein yields (Table 2) and enzyme activities (Table 3), it can be inferred that the syntheses of cellulolytic enzymes and xylanase by the fungus were greatly enhanced in CBR medium compared to those in CBAX1 or CBAX2 medium. This inference was confirmed by fungal secretome analysis (see below).

Among these protein blends, E_CBAX1 had the highest saccharification efficiency on the extractable arabinoxylans, with the following optimal reaction conditions: pH of 4.0, temperature of 55 °C, and reaction time of 72 h. CBAX, one of the most complex plant xylans with a high branching degree and various substitutions, exhibited remarkable resistance to enzymatic hydrolysis by pure endo-xylanase as well as some commercial enzyme cocktails in previous reports [45, 46]. Here, E_CBAX1 (5.0% dosage) liberated the majorities of xylose (87.0–93.8%) and arabinose (80.8–85.8%) in CBAX1 or CBAX2 (Additional file 3: Tables S2–S4). Meanwhile, low conversion ratios (11.8–17.8%) of pentose on these substrates were observed when using the same dosage of CTec3 (Additional file 3: Table S3), a commercial enzyme cocktail with highly efficient cellulases and hemicellulases (https://biosolutions.novozymes.com/). Complete monosaccharification of CBAX depends on the synergistic actions of multiple enzymes [10]. We speculated that a relatively integral CBAX-degrading enzyme system existed in E_CBAX1 due to its high efficiency in saccharification of the substrate. Meanwhile, the proportion of enzymes involved in CBAX degradation in the protein blend is presumably imbalanced because the high efficiency of enzymatic hydrolysis was dependent on high enzyme usage. Almost complete saccharification of CBAX1 and CBAX2 was achieved by combining E_CBAX1 and CTec3 (Additional file 3: Tables S3 and S4), suggesting the functional complementarity of the two enzyme mixtures on the decomposition of the substrates. Further, clarifying the key components of the CBAX-degrading enzyme system is required for outstanding saccharification of the substrate with a low enzyme dosage. Additionally, the same amount of E_CBAX1 (especially at a low enzyme dosage) had a lower conversion ration of pentose on CBAX1 than that on CBAX2 (Additional file 3: Table S2). One possible explanation is that more recalcitrant structures were contained in the former than in the latter. Further research on the substrate structures is required to well understand the difference in enzymatic hydrolysis of the two xylans.

The CAZyme repertoire of *P.* *parvum* was investigated by genome sequencing as well as secretome analysis. According to the genome (25.8 Mb) annotation, the fungus possesses abundant CAZymes (376) responsible for the decomposition, biosynthesis, or modification of glycoconjugates, oligosaccharides, and polysaccharides [47]. The number and diversity of CAZyme-encoding genes in *P.* *parvum* are comparable with many famous lignocellulose-degrading fungi (Additional file 7: Table S11), such as *P.* *decumbens* [48], *T.* *reesei* [49], and *A.* *niger* [50]. As expected, many proteins involved in lignocellulose degradation were found in the fungal CAZyme repertoire, including complete cellulose-degrading enzymes and multiple enzymes involved in the depolymerisation of heteroxylans (Additional file 8: Table S12). Meanwhile, six previously characterised lignocellulolytic enzymes were detected in the predicted CAZyme repertoire: two endo-β-1,4-xylanases belonging to GH10 (PP06205) or GH11 (PP00661) families [32], two GH62 α-l-arabinofuranosidases (PP07503; PP08284) [30, 31], a CE1 feruloyl esterase (PP07504) [51], and an AA9 family LPMO (PP08607) [52].

Approximately 41% (108) or 37% (118) of the proteins were identified as CAZymes in the secretome of *P.* *parvum* grown on CBAX1 or CBR, respectively (Table 5). Eighteen CAZymes without a putative SP at their N-terminus appeared in the fungal secretomes (Additional file 9: Table S13). The same phenomenon was reported in the secretomes of *P.* *oxalicum* [53] and could be explained as protein secretion via non-classical secretory mechanism(s), protein leakage during cell autophagy, or autolysis [53]. The composition of CAZymes in the fungal secretome differed between the two carbon sources. Using CBAX1 as a carbon source, the fungus mainly synthesised hemicellulolytic enzymes (36.8% of secretome), particularly GH3 β-1,4-xylosidase, GH30_7 endo-β-1,4-xylanase, GH51 and GH54 α-l-arabinofuranosidases, and GH27 α-galactosidase (Fig. 6a; Table 6). Among cellulolytic enzymes, only GH3 β-1,4-glucosidase was abundantly produced by the fungal strain under the induction conditions. In contrast, CBR induced the synthesis of abundant cellulolytic enzymes (37.1% of the secretome) in *P.* *parvum*, including β-1,4-glucosidase (GH3), endo-β-1,4-glucanase (GH5, 7, and 12), cellobiohydrolase (GH6 and 7), and LPMO (AA9). High levels of hemicellulolytic enzymes (24.7% of the secretome) were simultaneously produced by the fungus in the presence of the complex substrate (Fig. 6a; Table 6).

When exposed to lignocellulosic biomass, many filamentous fungi can secrete a small amount of lignocellulolytic enzymes under the induction of carbon starvation [54, 55]. These enzymes degrade plant polysaccharides into low molecular sugars (e.g., cellobiose, xylobiose, d-xylose, and l-arabinose), which are natural inducers of cellulase and/or hemicellulase genes in different filamentous fungi [56]. In the present study, the inducers derived from arabinoxylan (CBAX1) did not activate the expression of cellulases (except GH3 β-1,4-glucosidase) in *P.* *parvum*. Meanwhile, a variety of inducers from CBR (cellulose and arabinoxylan) led to high expression of cellulase as well as hemicellulase genes in the fungus. Of note, the production level of endo-β-1,4-xylanases (two GH10, one GH11, and two GH30) in the fungus under the induction of CBR was higher than that of CBAX1 (Table 6; Fig. 6c). Similarly, Liao et al. reported that a combined carbon source (cellulose and xylan) strongly enhanced the production of xylanase in *P.* *oxalicum* [57]. A possible explanation is that positive synergistic effects existed in enzyme induction between cellulose and xylan when the fungus was grown on the substrate [57]. In addition, *P.* *parvum* 4-14 exhibited a great capability of producing glycosidases, including β-xylosidase, β-glucosidase, α-galactosidase, and α-l-arabinofuranosidase, reflecting that up to 37.2% of the secretome (E_CBAX1) was made up of the four enzymes (Fig. 6b). These glycosidases have important potential for the complete saccharification of lignocellulosic biomass [10, 58].

A series of enzymes involved in decomposition of plant xylans were identified in the fungal secretomes, including seven endo-β-1,4-xylanases (GH10, 11, or 30), two β-1,4-xylosidase (GH3), five α-l-arabinofuranosidases (GH43, 51, 54, or 62), one α-1,2-glucuronidase (GH67), two feruloyl esterases (CE1), and two acetyl xylan esterases (CE5 or 16) (Table 6). These enzymes are responsible for the breakdown of the β-1,4-linked xylose backbone or the removal of simple substitutions (e.g., Ara*f* and m-GlcA) from xylan [10]. Importantly, the backbone of CBAX is decorated by not only simple substitutions but also complex oligosaccharide side chains (typically FAXG) (Additional file 1: Fig. S1). These oligosaccharide substitutions are resistant to enzymatic hydrolysis and greatly limit the saccharification of CBAX [17, 18]. In the current study, high-level saccharification of CBAX1 and CBAX2 was achieved by E_CBAX1, suggesting that the fungal secretome contained special enzymes acting on the oligosaccharide side chains in the xylans. For example, the four putative GH27 α-galactosidases existing in the fungal secretomes might cleave α-l-galactopyranosyl moieties in the FAXG structure. Additionally, the functional complementarity of *P.* *parvum* secretomes and the enzyme cocktail CTec3 (designed based on *T.* *reesei* lignocellulolytic enzymes) was observed in the saccharification experiments, indicating that one or several components of the latter played an important role in the decomposition of CBAX. For example, *T.* *reesei* instead of *P.* *parvum* can synthesise GH115 α-glucuronidase (Additional file 8: Table S12), which may facilitate the removal of the m-GlcA substituent from CBAX. Further research is needed to clarify the key enzymes involved in the breakdown of CBAX (particularly the large side chains) and explain the mechanism of synergistic biodegradation of the complex substrate.

## Conclusions

The secretomes of *P.* *parvum* 4-14 (E_CBAX1, E_CBAX2, or E_CBR) were prepared by culturing the fungus on different fractions (CBAX1, CBAX2, or CBR) of AHP-pretreated corn bran. Under the optimal conditions, the fungal secretome E_CBAX1 effectively released monosaccharides (xylose, arabinose, and glucose) from CBAX1 and CBAX2, with a conversion ratio greater than 80%. Almost complete saccharification of the substrates was achieved by combining E_CBAX1 and the enzyme cocktail CTec3. The fungal secretome E_CBR effectively released glucose (89.3%), xylose (64.2%), and arabinose (64.1%) from CBR. The combination of E_CBR and CTec3 enhanced saccharification of the solid substrate, and the conversion ratios of glucose, xylose, and arabinose were up to 97.3%, 81.4%, and 76.1%, respectively. Genomic analysis indicated that the fungus possesses 376 putative CAZymes, including plentiful lignocellulolytic enzymes. Proteomic analysis confirmed that complete cellulases and a variety of hemicellulases with differential expression profiles were produced by the fungus grown on CBAX1 or CBR. High levels of glycosidases (e.g., β-xylosidase, β-glucosidase, α-galactosidase, and α-L-arabinofuranosidase) were secreted by the fungus under the growth conditions. The fungal lignocellulolytic enzymes exhibited promising potential for the monosaccharification of AHP-pretreated corn bran.

## Methods

### Biomaterials, medium and substrates

*P.* *parvum* 4-14 (CCTCC M2015404) was maintained on potato dextrose agar (PDA) slants at 4 °C in our lab [33]. The (hemi-)cellulase cocktail CTec3 was provided by Novozymes (A/S, Bagsvaerd, Denmark). Mandels’ medium [59] with minor modifications was used for fungal fermentation, which contained 10 g/L glucose (or other carbon sources), 2 g/L KH_2_PO_4_, 1.4 g/L (NH_4_)_2_SO_4_, 0.3 g/L urea, 0.3 g/L MgSO_4_·7H_2_O, 0.3 g/L CaCl_2_, 0.3 g/L yeast extract, and 1 mL salt solution (CoCl_2_·6H_2_O 3.7 g/L, ZnSO_4_·7H_2_O 1.4 g/L, MnSO_4_·H_2_O 1.6 g/L, FeSO_4_·7H_2_O 5.0 g/L), with pH 5.0. The artificial substrates *p*-nitrophenyl-β-d-glucopyranoside (*p*NPGlu), *p*-nitrophenyl-β-d-xylopyranoside (*p*NPXyl), and *p*-nitrophenyl-α-d-galactopyranoside (*p*NPGal) were ordered from Macklin Biochemical Co., Ltd. (Shanghai, China), and *p*-nitrophenyl-α-l-arabinofuranoside (*p*NPAra*f*) was purchased from Megazyme Co., Ltd. (Wicklow, Ireland). PASC was prepared according to a previous method [60]. Corn bran was provided by Rongsheng Biotech. Co., Ltd. (Nanyang, China).

### Pretreatment of corn bran and chemical analyses

Corn bran was treated with amylase and papain to remove starch and protein, as described previously [51]. Arabinoxylans were extracted from the destarched corn bran by AHP method [39, 61]. Briefly, 50 g of raw material was treated with 1 L of 2% H_2_O_2_ solution with pH 11.5 (adjusted with solid NaOH) at 40 °C for 24 h. After solid–liquid separation by vacuum filtration with a microporous membrane (0.45 μm), arabinoxylans in the soluble fraction were subjected to graded precipitation by adding ethanol to the final concentrations of 50% and 75%. The arabinoxylans obtained by precipitation with low and high ethanol concentrations were named CBAX1 and CBAX2, respectively. The solid fraction (CBR) was washed three times with distilled water and dried to constant weight in an oven with 60 °C.

The structural carbohydrate and lignin contents in the raw material, extracted arabinoxylans, and solid residue were determined using the two-step acid hydrolysis method [62]. The monosaccharides released from the substrates by acid hydrolysis were quantified via high-performance anion-exchange chromatography (Dionex ICS-5000, Sunnyvale, CA, USA) coupled with a CarboPac PA10 analytical column (250 mm × 2 mm, Dionex) and a pulsed amperometric detector, as reported previously [63]. The acid-insoluble (Klason) lignin was isolated from the hydrolysate by filtration, dried at 105 °C and weighed [30].

### Fungal fermentation for enzyme production

*P.* *parvum* 4-14 was grown on a PDA plate at 28 °C for 1 week. To prepare seed culture, a fungal block was aseptically transferred into a 250-mL Erlenmeyer flask containing 50 mL of Mandels’ medium with glucose as the carbon source and incubated on a rotary shaker set at 200 rpm and 28 °C for 120 h. Two millilitres of the fungal seed culture were transferred into a 250-mL flask containing 50 mL of 2 × Mandels’ medium with CBAX1, CBAX2, or CBR as the carbon source. Fermentation was carried out on a rotary shaker set at 28 °C and 200 rpm for 192 h. All fermentation experiments were performed in triplicate. The fermentation broth was sampled periodically and centrifuged at 12,000×*g* for 10 min. The total protein in the supernatant was quantified using a BCA assay kit (Thermo Tech, USA).

After fermentation, the supernatant was separated from the fungal mycelium and insoluble substances by vacuum filtration. Mycelial biomass was measured after drying to constant weight at 70 °C. To concentrate the protein solution, the crude extract was subjected to salting-out and dialysis treatments. Briefly, 24 g of ammonium sulphate powder was slowly mixed with 40 mL of the crude extract with continuous stirring, resulting in a salt concentration of 60%. Proteins in the salt solution were precipitated overnight at 4 °C, separated from the liquid fraction by centrifugation at 10,000×*g* for 10 min, and re-dissolved in 4 mL phosphate buffered saline (137 mM NaCl, 2.7 mM KCl, 10 mM Na_2_HPO_4_, 2 mM KH_2_PO_4_, pH 7.4). After the same centrifugation, inorganic salts, monosaccharides, and oligosaccharides in the supernatant were removed by dialysis (3 kDa cutoff) against 500 mL phosphate buffer (0.1 M, pH 8.0) for 24 h (the buffer was changed every 8 h). Protein samples were quantified using a BCA assay kit and analysed using SDS-PAGE.

### Enzymatic activity assay

The activities of crude xylanase, endoglucanase (CMCase), and cellulase were determined using beechwood xylan (Sigma, St. Louis, MO, USA), carboxymethyl cellulose (CMC-Na) (Sigma, St. Louis, MO, USA), and PASC as substrates, respectively. The reaction system consisted of 500 μL sodium acetate (0.1 M, pH 5.0) buffer, 50 μL (about 1 μg) enzyme sample, and 450 μL substrate solution (2% CMC-Na, 0.5% beech wood xylan, or 1% PASC). After reaction at 50 °C for 10 min, the reducing sugar released from the corresponding substrate was quantified by the 3,5-dinitrosalicylic acid (DNS) method [64] using a glucose or xylose standard curve. The activity assays of β-glucosidase, α-l-arabinofuranosidase, β-xylosidase, or α-galactosidase were carried out using *p*NPGlu, *p*NPAra*f*, *p*NPXyl, or *p*NPGal as substrates, respectively. The reaction system contained 50 μL sodium acetate buffer (0.1 M, pH 5.0), 5 μL substrate (20 mM), 10 μL enzyme (50 ng/μL), and 35 μL dH_2_O. The mixture was incubated at 50 °C for 10 min, and the reaction was quenched by adding 400 μL of 0.2 M NaCO_3_ solution. The released *p*-nitrophenol was quantified by measuring the absorbance at *OD*_405nm_ and calculating the concentration using a standard curve. One unit (U) of enzyme activity was defined as the amount of enzyme liberating one μmol of glucose, xylose, or *p*-nitrophenyl from the corresponding substrate per minute under the assay conditions.

### Saccharification experiments

Enzyme hydrolysis of soluble arabinoxylans (CBAX1 and CBAX2) was performed in 2-mL centrifuge tubes. Two hundred microlitres reaction system contained 0.1 M sodium acetate buffer (pH 5.0), 4 mg substrate, and 25–200 μg protein sample. The mixture was incubated at 50 °C and 1000 rpm in a Thermomixer for 48 h (or the set time), and then treated at 99 °C for 10 min to stop the reaction. Different reaction conditions involving pH, temperature, reaction time, enzyme dosage, and metal ion supplementation were designed in the enzymatic hydrolysis experiments. The amounts of total reducing sugars released from the substrates were determined by DNS method. The monosaccharides (glucose, xylose, and arabinose) in the hydrolysates were quantified by high-performance liquid chromatography (HPLC) (Agilent 1100, Palo Alto, CA, USA) equipped with a Bio-Rad Aminex HPX-87 H column and a refractive index detector, as reported previously [29].

CBR was hydrolysed by the fungal CAZymes alone or in combination with the enzyme cocktail CTec3. In a 2-mL centrifuge tube, 100 mg substrate was mixed with 0.75 mL sodium acetate buffer (0.1 M, pH 5.0), 200–600 μg crude enzymes, 20 μL tetracycline hydrochloride solution (4 mg/mL) as an antimicrobial reagent, and dH_2_O to 1 mL. The hydrolysis reaction was carried out at 50 °C in a Thermomixer for 72 h. The monosaccharides liberated from the substrates were quantified by HPLC analysis. All hydrolysate measurements were performed at least in triplicate, and mean values are presented. Conversion ratios (%) of glucose, xylose, or arabinose on the substrate were calculated as follows:

$$\mathrm{Conversion\,ratio }\left(\%\right)=\frac{\mathrm{amount\,of\,released\,monosaccharide\,by\,enzymatic\, hydrolysis}}{\mathrm{amount\,of\,monosaccharide\,in\,substrate}}.$$

### Genome sequencing, assembly and annotation

*P.* *parvum* 4-14 was grown on a PDA plate at 37 °C for 7 days, and fungal ascospores were collected using a previously described method [65]. Single-ascospore isolates were obtained through serial dilution and spread on PDA plates. A pure isolate (numbered 4-14b) was selected and maintained on a PDA slant for genomic sequencing. After propagation in liquid PDA medium, the fungal isolate was subjected to genomic DNA extraction using the standard cetyltrimethylammonium bromide method [66]. Whole-genome sequencing of the fungal isolate was performed by the Beijing Novogene Bioinformatics Technology Co., Ltd (Beijing, China) using the PacBio Sequel platform (continuous long read mode) in combination with the Illumina NovaSeq PE150 platform, as detailed in a previous study [67]. The long reads from PacBio sequencing were corrected by the short reads from the Illumina platform and were assembled into contigs using SMRT Link v5.0.1 software [68]. Genome completeness was evaluated using BUSCO (version 5.4.3) software with the lineage dataset fungi_odb9 [69].

Gene annotation of the fungal genome was carried out using a whole-genome BLAST search (*E*-value less than 1e−5, minimal alignment length percentage larger than 40%) against the following databases: GO, KEGG, COG, NR, TCDB, Pfam, cytochrome P450, and Swiss-Prot. The CAZyme-encoding genes in the genome were analysed by searching the fungal proteins against the CAZymes database using dbCAN HMMdb v10.0 (https://bcb.unl.edu/dbCAN2/blast.php) [70]. Meanwhile, the secretory protein-encoding genes and secondary metabolism gene clusters in the fungal genome were predicted using SignalP 6.0 [71] in combination with TMHMM 2.0 (https://services.healthtech.dtu.dk) and antiSMASH 4.02 [72], respectively.

### Proteomic analysis

#### Filter-aided sample preparation

Label-free quantitative (LFQ) proteomics identification was employed to analyse the secreted proteins of *P.* *parvum* 4-14 after FASP digestion, as described previously [73, 74]. Fifty microlitres of protein sample (4 μg/μL) was blended with the same volume of 200 mM dithiothreitol solution and boiled for 5 min. After cooling to room temperature, 200 µL UA buffer (8 M Urea, 150 mM Tris–HCl, pH 8.0) was added to the protein solution twice to remove dithiothreitol and other small molecular components by ultrafiltration (10 kDa cutoff). To block reduced cysteine residues, 100 µL iodoacetamide (100 mM in UA) buffer were mixed with the sample at 600 rpm for one minute, incubated at 25 °C for 30 min in the dark, and centrifuged at 14,000×*g* for 15 min. The filter was washed with 100 µL of UA buffer twice and then 100 µL of 25 mM NH_4_HCO_3_ solution twice. The protein samples were digested with 4 μg of trypsin (Promega, Madison, USA) in 40 μL 100 mM NH_4_HCO_3_ at 37 °C for 18 h. The digested peptides were collected from the filter by centrifugation at 14,000×*g* for 15 min. The peptide solution was desalted with a C18 Cartridge (Sigma, Burlington, USA), freeze-dried, re-dissolved in 40 μL formic acid (0.1%) solution, and quantified by measuring the absorbance at *OD*_280nm._

#### LC–MS/MS analysis

LC–MS/MS analysis was employed to identify the peptide products. The peptides were separated using an EASY-nLC 1200 UHPLC system (Thermo Scientific, USA) equipped with a reverse phase trap column (100 μm × 2 cm, nanoViper C18 column) and a C18-reversed phase analytical column (75 μm × 100 mm, 3 μm particle size). The columns were balanced with 95% A buffer (0.1% formic acid) and eluted with a linear gradient of B buffer (0.1% formic acid and 84% acetonitrile) at a flow rate of 300 nL/min, which included 0–35% B buffer for 0–50 min, 35–100% B buffer for 50–55 min, and 100% B buffer for 55–60 min. The separated peptides were analysed using a Q-Exactive Mass spectrometer (Thermo Scientific, USA).

MS analysis for 90 min with a survey scan (300–1800 mass-to-charge ratios, *m*/*z*) was performed in the positive ion mode at a resolution of 70,000 at 200 *m*/*z*, automatic gain control target of 3 × 10^6^, and maximum injection time of 50 ms. The data (*m*/z) of peptides and peptide fragments were acquired as follows: 20 fragment files were collected after a full scan, higher-energy collision dissociation (HCD) fragmentation, 2 *m*/*z* of isolation window, 17,500 resolutions at 200 *m*/*z* of HCD spectra, 30 eV of normalised collision energy, 60.0 s of dynamic exclusion, and 0.1% of the underfill ratio. To identify and quantify proteins, the raw file was searched against the protein database of *P.* *parvum* 4-14 using MaxQuant software (v1.5.3.17) (www.maxquant.org/), with a false discovery rate ≤ 0.01. Three biological replicates were carried out, and the proteins with at least two unique peptides detected were selected for quantification and differential expression studies. Protein abundance was calculated on the basis of the normalised spectral protein intensity (LFQ intensity).

## Supplementary Information

**Additional file 1**: **Figure S1.** Structural model of corn bran arabinoxylan.

**Additional file 2**: **Figure S2. **Saccharification of CBAX1and CBAX2by different enzyme blends of *P.* *parvum* 4-14. **Figure S3.** Effect of metal ions on saccharification of CBAX1and CBAX2by E_CBAX1.

**Additional file 3**: **Table S1.** Monosaccharide liberation from CBAX by the enzyme blend E_CBAX1 of *P.* *parvum* at different reaction times. **Table S2.** Saccharification of CBAX by the enzyme blend E_CBAX1 of *P.* *parvum* at different dosages. **Table S3.** Saccharification of corn bran arabinoxylan 1by the enzyme blend E_CBAX1 of *P.* *parvum* and/orcellulase cocktail CTec3. **Table S4.** Saccharification of corn bran arabinoxylan 2by the enzyme blend E_ CBAX1 of *P.* *parvum* and/orcellulase cocktail CTec3. **Table S5.** Saccharification of CBR by two enzyme blends from *P.* *parvum* orcellulase cocktail CTec3 at different dosages. **Table S6.** Saccharification of CBR by the enzyme blend E_CBR of *P.* *parvum* and/orcellulase cocktail CTec3.

**Additional file 4**: **Table S7.** List of Illumina and single molecule real-timesequencing data of *P.* *parvum* 4-14. **Table S8.** General genome properties of *P.* *parvum* and other lignocellulose-degrading fungi. **Table S9.** The predicted secondary metabolism gene clusters in *P.* *parvum* 4-14.

**Additional file 5**: Sequences of predicted proteins in *P.* *parvum*.

**Additional file 6**: **Table S10.** The predicted CAZymes in *P.* *parvum* genome.

**Additional file 7**: **Table S11.** Comparison of the number of putative CAZyme-encoding genes in the genomes of five fungal species.

**Additional file 8**: **Table S12.** Comparison of the number ofcellulose-degrading enzymes among the three fungal species.

**Additional file 9**: **Table S13.** Summary of the identified proteins in the secretomes of *P.* *parvum* 4-14 grown on corn bran arabinoxylan1 or corn bran residue.

**Additional file 10:** **Figure S4.** Comparison of the numbers of main lignocellulolytic genesin the genome and the secretomes of *P.* *parvum*.

## Data Availability

All data generated or analyzed during this study are included in this published article and its supplementary information files.
