# Supplementary material for: Insights into the capability of the lignocellulolytic enzymes of Penicillium parvum 4-14 to saccharify corn bran after alkaline hydrogen peroxide pretreatment
Source: Biotechnol Biofuels Bioprod. 2023 May 11;16:79. doi: 10.1186/s13068-023-02319-x (PMC10176746; doi:10.1186/s13068-023-02319-x)
Supplement: Supplementary file 1 — Additional file 1: Figure S1. Structural model of corn bran arabinoxylan. [file 13068_2023_2319_MOESM1_ESM.docx]

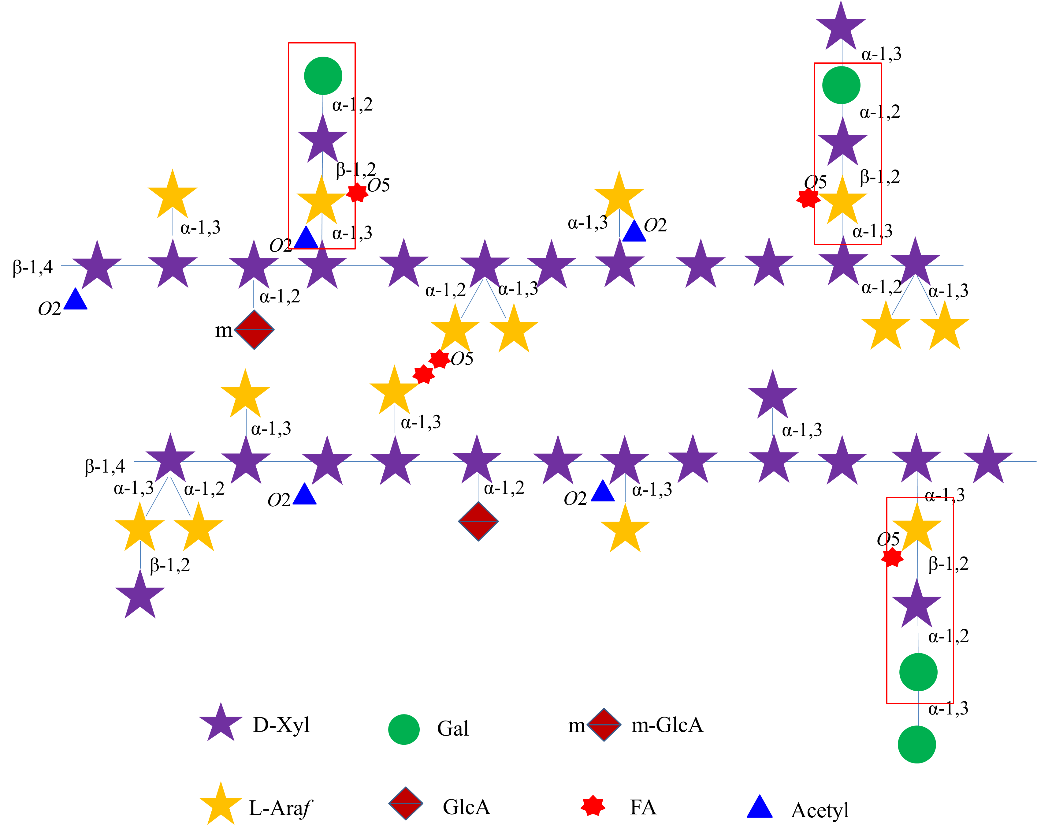


**Figure S1** Structural model of corn bran arabinoxylan [1,2]. D-Xyl, D-xylose residue; L-Ara*f*, L-Arabinose residue; Gal, Galactose residue; GlcA, α-D-glucuronic acid; m-GlcA, 4-*O*-methyl-α-D-glucuronic acid; FA, Ferulic acid; α-L-galactopyranosyl-(1,2)-β-D-Xyl*p*-(1,2)-5-*O*-*trans*-feruloyl-L-Ara*f* (FAXG) structures were marked with red box

**References:**

1. Allerdings E, Ralph J, Steinhart H, Bunzel M. Isolation and structural identification of complex feruloylated heteroxylan side-chains from maize bran. Phytochemistry. 2006;67(12):1276-1286. doi:10.1016/j.phytochem.2006.04.018

2. Rudjito RC, Jiménez-Quero A, Hamzaoui M, Kohnen S, Vilaplana F. Tuning the molar mass and substitution pattern of complex xylans from corn fibre using subcritical water extraction. Green Chem. 2020;22(23):8337-8352. doi:10.1039/d0gc02897e
