# Supplementary material for: Insights into the capability of the lignocellulolytic enzymes of Penicillium parvum 4-14 to saccharify corn bran after alkaline hydrogen peroxide pretreatment
Source: Biotechnol Biofuels Bioprod. 2023 May 11;16:79. doi: 10.1186/s13068-023-02319-x (PMC10176746; doi:10.1186/s13068-023-02319-x)
Supplement: Supplementary file 2 — Additional file 2: Figure S2. Saccharification of CBAX1and CBAX2by different enzyme blends of P. parvum 4-14. Figure S3. Effect of metal ions on saccharification of CBAX1and CBAX2by E_CBAX1. [file 13068_2023_2319_MOESM2_ESM.docx]

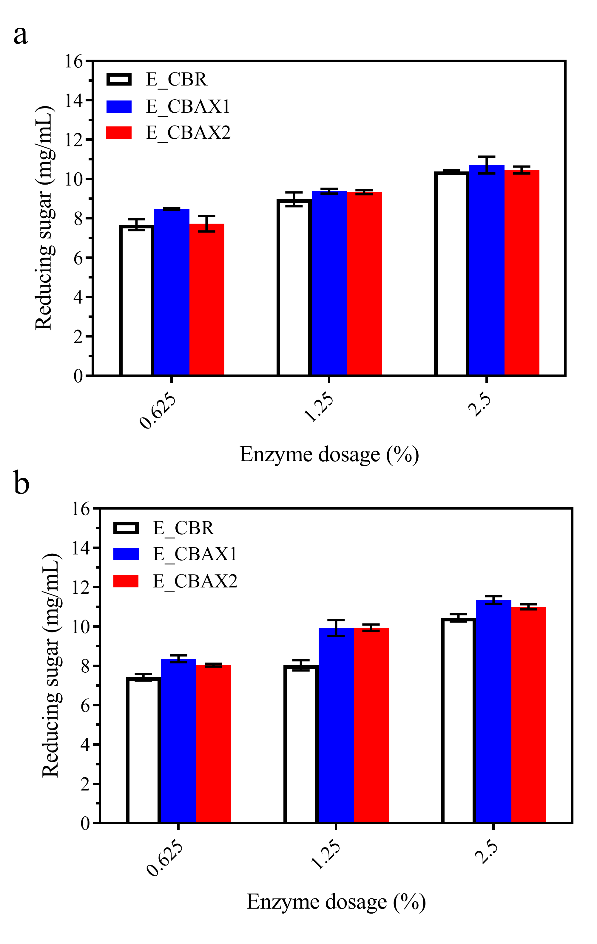


**Figure S2** Saccharification of CBAX1 (a) or CBAX2 (b) by different enzyme blends of *P. parvum* 4-14. The reaction system contained 200 μL sodium acetate buffer (0.1 M, pH 5.0), 4 mg substrate, and 25-100 μg enzymes. After incubation at 50^o^C for 48 h, the released reducing sugars were quantified by DNS method. E_CBR or E_CBAX1(or 2), the enzyme blend produced by the fungus using corn bran residue (CBR) or corn bran arabinoxylans (CBAX) 1 (or 2) as the sole carbon source. Error bars represent standard deviations from three repeated determinations


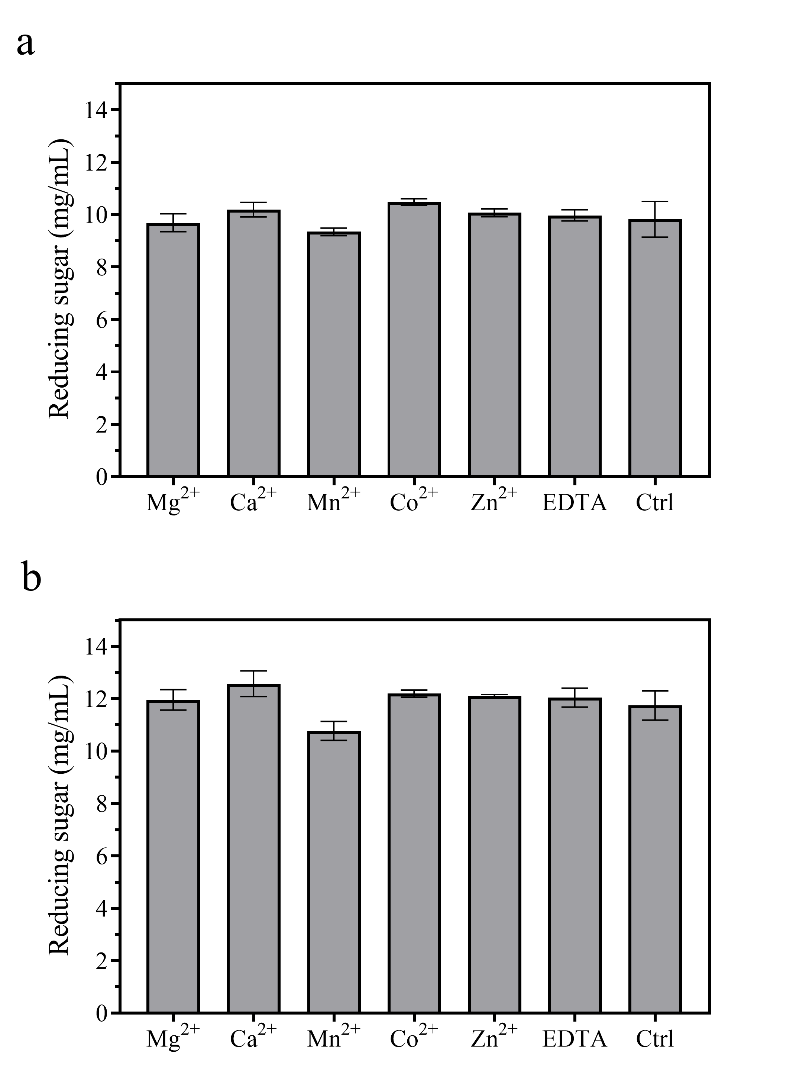


**Figure S3** Effect of metal ions on the saccharification of CBAX1 (**a**) and CBAX2 (**b**) by E_CBAX1. The hydrolysis reaction was carried out in 200 μL sodium acetate buffer (0.1 M, pH 4.0) containing 4 mg CBAX1 (or CBAX2) and 50 μg enzymes, 1 mM metal ions or EDTA. After incubation at 50^o^C for 48 h, the released reducing sugars were quantified by DNS method. E_CBAX1, the enzyme blend produced by *P. parvum* 4-14 using corn bran arabinoxylans (CBAX) 1 as the sole carbon source. Ctrl, no additional metal ions and ETDA. Error bars represent standard deviations from three repeated measurements
