# Supplementary material for: Insights into the capability of the lignocellulolytic enzymes of Penicillium parvum 4-14 to saccharify corn bran after alkaline hydrogen peroxide pretreatment
Source: Biotechnol Biofuels Bioprod. 2023 May 11;16:79. doi: 10.1186/s13068-023-02319-x (PMC10176746; doi:10.1186/s13068-023-02319-x)
Supplement: Supplementary file 3 — Additional file 3: Table S1. Monosaccharide liberation from CBAX by the enzyme blend E_CBAX1 of P. parvum at different reaction times. Table S2. Saccharification of CBAX by the enzyme blend E_CBAX1 of P. parvum at different dosages. Table S3. Saccharification of corn bran arabinoxylan 1by the enzyme blend E_CBAX1 of P. parvum and/orcellulase cocktail CTec3. Table S4. Saccharification of corn bran arabinoxylan 2by the enzyme blend E_ CBAX1 of P. parvum and/orcellulase cocktail CTec3. Table S5. Saccharification of CBR by two enzyme blends from P. parvum orcellulase cocktail CTec3 at different dosages. Table S6. Saccharification of CBR by the enzyme blend E_CBR of P. parvum and/orcellulase cocktail CTec3. [file 13068_2023_2319_MOESM3_ESM.docx]

**Table S1** Monosaccharide liberation from CBAX by the enzyme blend E_CBAX1 of *P. parvum* at different reaction times

| Time (h) | Released monosaccharide (mg/g substrate) | | | |  | Conversion ratio (%) | | |
| --- | --- | --- | --- | --- | --- | --- | --- | --- |
|  | Glucose | Xylose | Arabinose | Total | A/X | Glucose | Xylose | Arabinose |
| Substrate CBAX1 | | | | | | | | |
| 2 | 99.9±0.6 | 116.8±0.9 | 88.8±1.1 | 305.5 | 0.76 | 84.0 | 28.5 | 39.4 |
| 6 | 107.4±1.5 | 168.5±2.5 | 114.5±2.1 | 383.7 | 0.68 | 84.7 | 41.1 | 50.8 |
| 12 | 100.8±3.5 | 195.7±3.2 | 119.2±2.0 | 415.7 | 0.61 | 84.7 | 47.8 | 52.9 |
| 24 | 104.7±2.6 | 246.2±2.9 | 135.9±2.9 | 483.5 | 0.55 | 85.2 | 60.1 | 60.3 |
| 48 | 102.2±1.8 | 256.6±11.3 | 137.4±1.7 | 496.2 | 0.54 | 85.9 | 62.6 | 61.0 |
| 72 | 101.7±4.3 | 277.1±5.4 | 140.8±4.3 | 519.6 | 0.51 | 85.5 | 67.7 | 62.5 |
| Substrate CBAX2 | | | | | | | | |
| 2 | 22.4±0.4 | 145.4±1.2 | 142.3±0.9 | 310.0 | 0.98 | 71.7 | 33.5 | 46.9 |
| 6 | 23.7±0.6 | 210.3±2.8 | 183.7±4.1 | 417.8 | 0.87 | 76.1 | 48.4 | 60.6 |
| 12 | 22.4±0.5 | 245.5±2.8 | 195.6±2.7 | 463.5 | 0.80 | 71.7 | 56.5 | 64.5 |
| 24 | 22.6±0.4 | 286.1±6.6 | 208.5±3.9 | 517.2 | 0.73 | 72.5 | 65.8 | 68.8 |
| 48 | 24.4±0.4 | 343.6±4.6 | 229.6±3.4 | 597.6 | 0.67 | 78.1 | 79.1 | 75.7 |
| 72 | 24.7±0.5 | 363.3±0.8 | 234.2±0.6 | 622.2 | 0.64 | 79.1 | 83.6 | 77.3 |

In a 2-mL tube, 4 mg substrate was mixed with 0.2 mL sodium acetate buffer (0.1 M, pH 4.0) containing 50 μg (1.25% dosage) of E_CBAX1. Hydrolysis reactions were performed at 55°C and 800 rpm for 2 to 72 h. The released sugars were quantified by HPLC analysis. Corn bran arabinoxylans (CBAX) 1 and 2 were extracted from corncob raw material by the AHP pretreatment and the graded ethanol precipitation as described in the “Methods” section. E_CBAX1 was prepared by the culture of *P. parvum* 4-14 in Mandels' medium using CBAX1 as the sole carbon source. A/X, the ratio of arabinose to xylose. The data are the averages of three repeated measurements

**Table S2** Saccharification of CBAX by the enzyme blend E_CBAX1 of *P. parvum* at different dosages

| Enzyme dosage (%) | Released monosaccharide (mg/g substrate) | | | |  | Conversion ratio (%) | | |
| --- | --- | --- | --- | --- | --- | --- | --- | --- |
|  | Glucose | Xylose | Arabinose | Total | A/X | Glucose | Xylose | Arabinose |
| Substrate CBAX1 | | | | | | | | |
| 1.25 | 99.5±2.2 | 263.8±12.5 | 151.0±8.9 | 514.3 | 0.57 | 83.6 | 64.4 | 67.0 |
| 2.50 | 100.9±7.2 | 331.0±13.9 | 184.7±18.3 | 616.7 | 0.56 | 84.8 | 80.8 | 81.9 |
| 3.75 | 102.1±7.3 | 345.4±17.2 | 182.5±9.4 | 630.0 | 0.53 | 85.8 | 84.3 | 81.0 |
| 5.00 | 103.2±5.6 | 356.3±7.8 | 182.2±5.8 | 641.8 | 0.51 | 86.7 | 87.0 | 80.8 |
| Substrate CBAX2 | | | | | | | | |
| 1.25 | 23.1±0.6 | 344.1±3.4 | 231.2±2.0 | 598.5 | 0.67 | 74.2 | 79.2 | 76.3 |
| 2.50 | 23.7±1.0 | 386.3±3.9 | 246.8±1.5 | 656.8 | 0.64 | 75.8 | 88.9 | 81.4 |
| 3.75 | 24.8±0.1 | 404.1±3.9 | 256.1±0.4 | 685.0 | 0.63 | 79.6 | 93.0 | 84.5 |
| 5.00 | 24.8±0.5 | 407.7±10.8 | 255.8±5.6 | 688.3 | 0.63 | 79.4 | 93.8 | 84.4 |

In a 2-mL tube, 4 mg substrate was mixed with 0.2 mL sodium acetate buffer (0.1 M, pH 4.0) containing 50 to 200 μg of E_CBAX1 (1.25%-5.0%, g protein/g substrate). Hydrolysis reactions were performed at 50 °C and 800 rpm for 72 hours. The released sugars were quantified by HPLC analysis. Corn bran arabinoxylans (CBAX) 1 and 2 and enzyme blend E_CBAX1were prepared as described in Table S1. A/X, the ratio of arabinose to xylose. The data are the averages of three repeated measurements

**Table S3** Saccharification of corn bran arabinoxylan 1 (CBAX1) by the enzyme blend E_CBAX1 of *P. parvum* and/or (hemi)cellulase cocktail CTec3

| Enzyme combination (dosage, %) | Released monosaccharide (mg/g substrate) | | | |  | Conversion ratio (%) | | |
| --- | --- | --- | --- | --- | --- | --- | --- | --- |
|  | Glucose | Xylose | Arabinose | Total | A/X | Glucose | Xylose | Arabinose |
| CTec3(2.5) | 89.7±6.0 | 38.7±2.2 | 32.8±2.3 | 161.2 | 0.85 | 75.4 | 9.5 | 14.5 |
| CTec3(5.0) | 92.8±5.7 | 48.5±1.1 | 40.1±0.5 | 181.4 | 0.83 | 78.0 | 11.8 | 17.8 |
| E_CBAX1(2.5) | 98.0±4.1 | 337.4±22.3 | 184.1±7.2 | 619.5 | 0.55 | 82.3 | 82.4 | 81.7 |
| E_CBAX1(5.0) | 100.1±8.5 | 361.4±21.8 | 191.7±7.9 | 653.2 | 0.53 | 84.2 | 88.2 | 85.1 |
| E_CBAX1(1.25)+CTec3(1.25) | 102.0±1.8 | 330.3±8.8 | 185.5±4.5 | 617.9 | 0.56 | 85.8 | 80.6 | 82.3 |
| E_CBAX1(2.5)+CTec3(2.5) | 100.5±2.7 | 340.6±6.7 | 184.4±3.9 | 625.5 | 0.54 | 84.4 | 83.2 | 81.8 |
| E_CBAX1(5.0)+CTec3(5.0) | 108.8±6.9 | 402.9±7.6 | 219.9±12.6 | 731.5 | 0.55 | 91.4 | 98.4 | 97.5 |

In a 2-mL tube, 4 mg CBAX1 was mixed with 0.2 mL sodium acetate buffer (0.1 M, pH 4.0) containing single or combined enzyme mixtures. Hydrolysis reactions were performed at 50 °C and 800 rpm for 3 days. The released sugars were quantified by the HPLC analysis. CTec3, commercial enzyme cocktail Cellic® CTec3; E_CBAX1, the enzyme blend produced by *P. parvum* 4-14 using CBAX1 as carbon source. A/X, the ratio of arabinose to xylose. The data are the averages from three repeated measurements

**Table S4** Saccharification of corn bran arabinoxylan 2 (CBAX2) by the enzyme blend E_ CBAX1 of *P. parvum* and/or (hemi)cellulase cocktail CTec3

| Enzyme combination (dosage, %) | Released monosaccharide (mg/g substrate) | | | |  | Conversion ratio (%) | | |
| --- | --- | --- | --- | --- | --- | --- | --- | --- |
|  | Glucose | Xylose | Arabinose | Total | A/X | Glucose | Xylose | Arabinose |
| CTec3(2.5) | 25.9±3.8 | 44.0±4.1 | 41.0±4.5 | 110.8 | 0.93 | 82.9 | 10.1 | 13.5 |
| CTec3(5.0) | 25.2±1.4 | 57.2±2.0 | 50.8±2.1 | 133.2 | 0.89 | 80.7 | 13.1 | 16.8 |
| E_CBAX1(2.5) | 27.2±0.9 | 373.0±2.8 | 246.0±6.3 | 648.0 | 0.66 | 87.3 | 86.1 | 81.2 |
| E_CBAX1(5.0) | 27.6±0.4 | 396.8±1.5 | 260.1±2.4 | 684.5 | 0.66 | 88.5 | 91.2 | 85.8 |
| E_CBAX1(1.25)+CTec3(1.25) | 27.7±2.4 | 360.0±20.3 | 250.3±16.0 | 638.0 | 0.70 | 88.7 | 82.7 | 82.6 |
| E_CBAX1(2.5)+CTec3(2.5) | 29.6±1.0 | 426.7±4.7 | 279.8±3.3 | 736.1 | 0.66 | 94.9 | 98.0 | 92.3 |
| E_CBAX1(5.0)+CTec3(5.0) | 30.9±0.8 | 429.1±6.4 | 287.3±6.4 | 747.3 | 0.67 | 98.9 | 98.6 | 94.8 |

The experimental conditions and analysis methods were the same as those described in Table S3

**Table S5** Saccharification of CBR by two enzyme blends from *P. parvum* or (hemi-)cellulase cocktail CTec3 at different dosages

| Enzyme (dosage, %) | Released monosaccharide (mg/g substrate) | | | |  | Conversion ratio (%) | | |
| --- | --- | --- | --- | --- | --- | --- | --- | --- |
|  | Glucose | Xylose | Arabinose | Total | A/X | Glucose | Xylose | Arabinose |
| CTec3(0.2) | 451.7±2.9 | 38.7±0.5 | 18.1±0.6 | 508.5 | 0.47 | 79.3 | 21.0 | 19.9 |
| CTec3(0.4) | 527.7±6.7 | 49.6±0.7 | 20.8±0.5 | 598.1 | 0.42 | 92.7 | 26.8 | 23.0 |
| CTec3(0.6) | 555.3±18.8 | 59.5±4.9 | 24.2±3.3 | 639.0 | 0.41 | 97.5 | 32.2 | 26.7 |
| E_CBR(0.2) | 385.9±0.3 | 92.9±0.8 | 56.3±1.3 | 535.2 | 0.61 | 67.8 | 50.3 | 62.1 |
| E_CBR(0.4) | 480.8±1.2 | 111.9±0.3 | 62.0±1.2 | 654.6 | 0.55 | 84.4 | 60.6 | 68.3 |
| E_CBR(0.6) | 508.4±0.4 | 118.4±0.3 | 58.2±14 | 685.0 | 0.49 | 89.3 | 64.2 | 64.1 |
| E_CBAX1(0.2) | 68.3±2.0 | 85.5±4.2 | 55.9±1.7 | 209.8 | 0.65 | 12.0 | 46.3 | 61.7 |
| E_CBAX1(0.4) | 88.1±3.3 | 101.1±0.3 | 57.6±0.3 | 246.8 | 0.57 | 15.5 | 54.8 | 63.5 |
| E_CBAX1(0.6) | 96.2±6.0 | 113.2±1.9 | 63.4±1.8 | 272.8 | 0.56 | 16.9 | 61.3 | 69.9 |

In a 2-mL tube, 100 mg substrate was mixed with 1 mL sodium acetate buffer (0.1 M, pH 4.5) containing different enzyme mixtures. Hydrolysis reactions were performed at 50 °C and 1,200 rpm for 4 days. The released sugars were quantified by the HPLC analysis. CBR, corn bran residues after AHP pretreatment; CTec3, commercial (hemi)cellulase Cellic® CTec3; E_CBAX1 and E_CBR, the enzyme blends produced by *P. parvum* 4-14 using corn bran arabinoxylans (CBAX) 1 or CBR as carbon source, respectively. A/X, the ratio of arabinose to xylose. The data are the averages from three repeated measurements

**Table S6** Saccharification of CBR by the enzyme blend E_CBR of *P. parvum* and/or (hemi)cellulase cocktail CTec3

| Enzyme combination (dosage, %) | Released monosaccharide (mg/g substrate) | | | |  | Conversion ratio (%) | | |
| --- | --- | --- | --- | --- | --- | --- | --- | --- |
|  | Glucose | Xylose | Arabinose | Total | A/X | Glucose | Xylose | Arabinose |
| CTec3(0.4%) | 548.3±5.4 | 68.5±1.4 | 27.2±0.4 | 644.1 | 0.40 | 96.3 | 37.1 | 30.0 |
| E_CBR(0.4%) | 468.2±14.6 | 125.7±3.2 | 64.4±1.0 | 658.3 | 0.51 | 82.2 | 68.1 | 71.0 |
| E_CBR(0.4%)+CTec3(0.1%) | 527.8±7.2 | 134.0±2.3 | 65.7±1.9 | 727.6 | 0.49 | 92.7 | 72.6 | 72.4 |
| E_CBR(0.4%)+CTec3(0.2%) | 521.6±3.8 | 131.7±2.0 | 63.7±0.1 | 717.0 | 0.48 | 91.6 | 71.3 | 70.3 |
| E_CBR(0.4%)+CTec3(0.3%) | 537.8±11.8 | 136.8±1.2 | 65.5±1.5 | 740.1 | 0.48 | 94.4 | 74.1 | 72.2 |
| E_CBR(0.4%)+CTec3(0.4%) | 552.8±6.7 | 140.8±1.6 | 67.5±0.7 | 761.1 | 0.48 | 97.1 | 76.3 | 74.4 |
| E_CBR(0.6%)+CTec3(0.6%) | 554.1±8.6 | 150.2±0.9 | 69.0±0.9 | 773.3 | 0.46 | 97.3 | 81.4 | 76.1 |

The experimental conditions and analytical methods were the same as those described in Table S7
