# Supplementary material for: Insights into the capability of the lignocellulolytic enzymes of Penicillium parvum 4-14 to saccharify corn bran after alkaline hydrogen peroxide pretreatment
Source: Biotechnol Biofuels Bioprod. 2023 May 11;16:79. doi: 10.1186/s13068-023-02319-x (PMC10176746; doi:10.1186/s13068-023-02319-x)
Supplement: Supplementary file 10 — Additional file 10: Figure S4. Comparison of the numbers of main lignocellulolytic genesin the genome and the secretomes of P. parvum. [file 13068_2023_2319_MOESM10_ESM.docx]

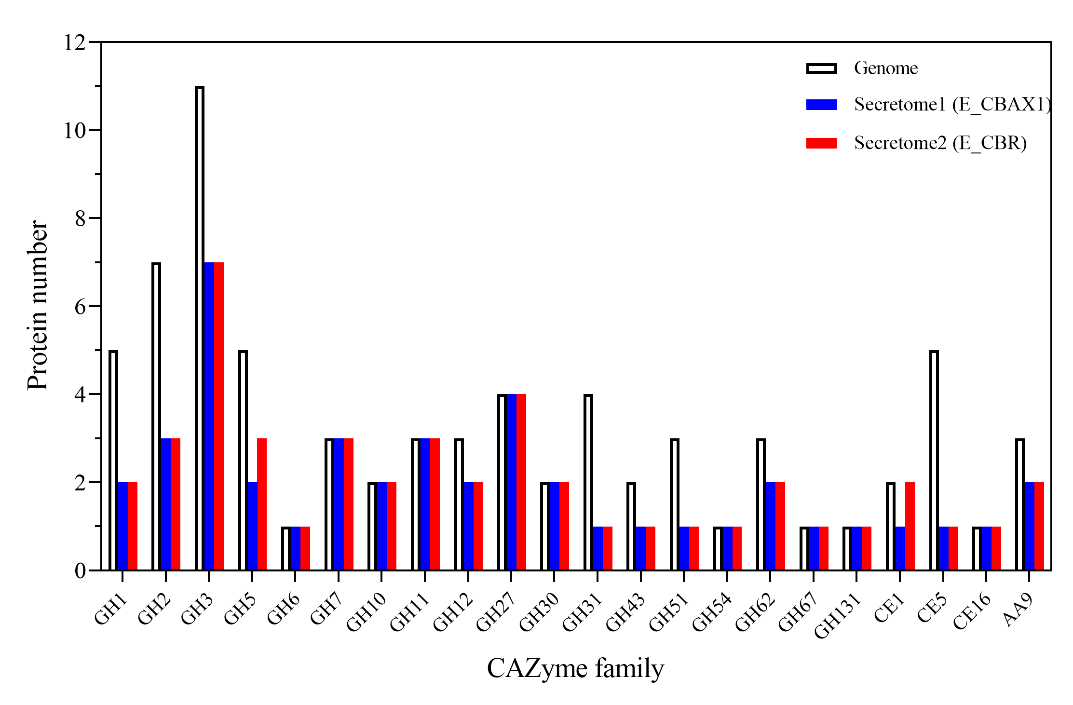


**Figure S4** Comparison of the numbers of main lignocellulolytic genes (enzymes) in the genome and the secretomes of *P. parvum*. E_CBAX1 and E_CBR, the proteins secreted by *P. parvum* 4-14 in the medium with CBAX1 or CBR as carbon source, respectively. Three GH1, three GH2, four GH3, two GH5, one GH27, two GH31, four GH43, one GH51, and one CE16 proteins did not contain predictable signal peptides (Additional file 6: Table S10). GH, glycoside hydrolase; CE, carbohydrate esterase; AA, auxiliary activity
